# Amyloid beta 42 alters cardiac metabolism and impairs cardiac function in male mice with obesity

**DOI:** 10.1038/s41467-023-44520-4

**Published:** 2024-01-15

**Authors:** Liam G. Hall, Juliane K. Czeczor, Timothy Connor, Javier Botella, Kirstie A. De Jong, Mark C. Renton, Amanda J. Genders, Kylie Venardos, Sheree D. Martin, Simon T. Bond, Kathryn Aston-Mourney, Kirsten F. Howlett, James A. Campbell, Greg R. Collier, Ken R. Walder, Matthew McKenzie, Mark Ziemann, Sean L. McGee

**Affiliations:** 1 Institute for Mental and Physical Health and Clinical Translation, Metabolic Research Unit, School of Medicine, Deakin University, Geelong, Australia; 2 Department of Cellular and Physiological Sciences, Faculty of Medicine, The University of British Columbia, Vancouver, Canada; 3 Becton Dickinson GmbH, Medical Affairs, 69126 Heidelberg, Germany; 4 Institute of Experimental Cardiovascular Research, University Medical Centre Hamburg-Eppendorf, Hamburg, Germany; 5 Institute for Physical Activity and Nutrition, School of Exercise and Nutrition Sciences, Deakin University, Geelong, Australia; 6 Department of Nutrition, Dietetics and Food, School of Clinical Sciences and Victorian Heart Institute, Monash University, Melbourne, Australia; 7 Baker Heart and Diabetes Institute, Melbourne, Australia; 8 Ambetex Pty Ltd, Geelong, Australia; 9 School of Life and Environmental Science, Deakin University, Geelong, Australia

**Keywords:** Obesity, Heart failure, Mechanisms of disease

## Abstract

There are epidemiological associations between obesity and type 2 diabetes, cardiovascular disease and Alzheimer’s disease. The role of amyloid beta 42 (Aβ_42_) in these diverse chronic diseases is obscure. Here we show that adipose tissue releases Aβ_42_, which is increased from adipose tissue of male mice with obesity and is associated with higher plasma Aβ_42_. Increasing circulating Aβ_42_ levels in male mice without obesity has no effect on systemic glucose homeostasis but has obesity-like effects on the heart, including reduced cardiac glucose clearance and impaired cardiac function. The closely related Aβ_40_ isoform does not have these same effects on the heart. Administration of an Aβ-neutralising antibody prevents obesity-induced cardiac dysfunction and hypertrophy. Furthermore, Aβ-neutralising antibody administration in established obesity prevents further deterioration of cardiac function. Multi-contrast transcriptomic analyses reveal that Aβ_42_ impacts pathways of mitochondrial metabolism and exposure of cardiomyocytes to Aβ_42_ inhibits mitochondrial complex I. These data reveal a role for systemic Aβ_42_ in the development of cardiac disease in obesity and suggest that therapeutics designed for Alzheimer’s disease could be effective in combating obesity-induced heart failure.

## Abstract (web summary)

Epidemiological evidence has identified associations among obesity, Alzheimer’s disease, and cardiovascular disease. Here, the authors report that adipose tissue releases amyloid beta 42 (Aβ42) and that antagonizing Aβ42 protects cardiac function in obesity murine models.

## Introduction

Epidemiological evidence has identified associations between obesity, Alzheimer’s disease (AD) and cardiovascular disease^1–3^. Common underlying aetiological factors such as inflammation, hypertension and hormonal alterations have all been implicated, however, the exact mechanisms involved remain largely unexplored. Another potential molecular link are amyloid beta peptides (Aβ), a putative pathogenic driver of AD^4^. Aβ peptides, ranging from 36-44 amino acids in length, are derived by proteolytic processing of the trans-membrane amyloid precursor protein (APP)^5^. Initial cleavage by either α- or β-secretases commits APP to the non-amyloidogenic or amyloidogenic pathways, respectively^5^. Following cleavage by the β-secretase BACE1, the remaining β-C-terminal fragment of APP can be cleaved by γ-secretase, which produces Aβ peptides that are released into the extracellular space via exocytosis^5^. Aβ peptides of 40 and 42 amino acids (Aβ_40_ and Aβ_42_) are the most common and Aβ_42_ has a particular propensity to aggregate and form oligomers^4^. The oligomerisation state of Aβ_42_, which is highly dynamic and stochastic, impacts its function and ability to interact with numerous cell surface receptors^6–8^. Extracellular Aβ_42_ can also be internalised and interact with organelles, intracellular signalling molecules and enzymes, disrupting normal cellular function^9^.

Accumulation of Aβ_42_ in the central nervous system is linked with alterations in metabolism, including impairments in glucose uptake^10,11^, glucose utilisation^12^ and aspects of mitochondrial function^13–16^ in several different cell types. APP and its proteolytic processing pathways are expressed in peripheral tissues and Aβ peptides are found in plasma^17^. The expression of APP is increased in adipose tissue of humans affected by obesity^18^ and APP overexpression in adipose tissue of mice causes adiposity and insulin resistance due to impaired adipocyte mitochondrial function^19^. Circulating concentrations of Aβ_42_ correlate with fat mass, however whether circulating Aβ_42_ regulates peripheral metabolism, particularly in the context of obesity, remains largely unexplored. This study aimed to determine whether Aβ_42_ is released by adipose tissue and whether this is increased in obesity. Finally, this study also sought to determine the metabolic consequences of persistently increased circulating levels of Aβ_42_. Here we show that adipose tissue releases Aβ_42_, which is increased in obesity and associates with higher plasma Aβ_42_. We also show that increases in plasma Aβ_42_ negatively impact cardiac metabolism and function and that Aβ_42_ accumulates in cardiac mitochondria and inhibits complex I of the respiratory chain. Antagonising Aβ_42_ with a neutralising antibody prevents obesity-induced defects in cardiac function and prevents further decline of cardiac function in established obesity. These findings enhance our understanding of the impact of obesity on the heart and provide a rationale for the repurposing of AD therapies for the treatment of heart failure.

## Results

### Aβ_42_ is released from adipose tissue, which is increased in obesity

**Fig. 1:**
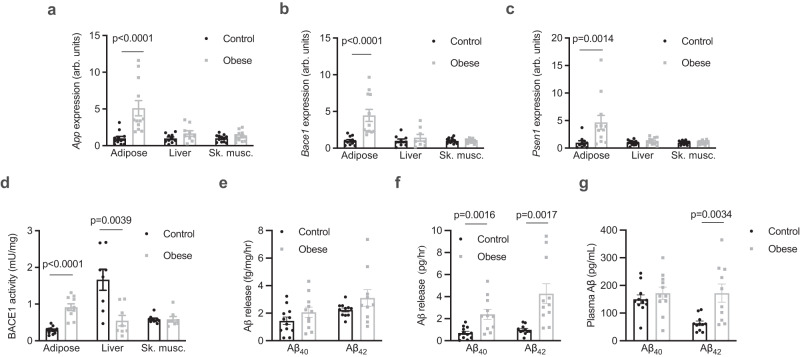
Ab_42_ is released from adipose tissue, which is increased in obesity. **a** *App* expression in adipose tissue (Mann Whitney test, U = 6; *n* = 12/group), the liver (*n* = 10 and 9/group respectively) and skeletal muscle (Sk. musc., quadriceps; unpaired t-tests; *n* = 12/group) from control and obese mice. **b** *Bace1* expression in adipose tissue (Mann Whitney test, U = 1; *n* = 12/group), the liver (*n* = 9 and 8/group, respectively) and sk. musc. (unpaired t-tests; *n* = 12/group) from control and obese mice. **c,** *Psen1* expression in adipose tissue (Mann Whitney test, U = 14; *n* = 10 and 12/group respectively), the liver (*n* = 12/group) and sk. musc. (*n* = 12/group) from control and obese mice (unpaired t-tests). **d** BACE1 activity in adipose tissue (*n* = 12 and 10/group respectively), the liver (*n* = 8/group) and sk. musc. (*n* = 8/group) from control and obese mice (unpaired t-tests). **e** relative release of Aβ isoforms from adipose tissue of control and obese mice normalised for tissue weight. **f** absolute release of Aβ isoforms from adipose tissue of control (*n* = 12) and obese (*n* = 11) mice (Mann Whitney test, U = 6). **g** plasma Aβ isoforms in control (*n* = 12) and obese (*n* = 11) mice (unpaired t-test). All data are mean ± SEM. Statistical tests are two-tailed. Source data are provided in the Source Data file.

To better understand the proteolytic processing of APP in the periphery in obesity, the expression and activity of APP and components of the amyloidogenic pathway were assessed in tissues from control and diet-induced obese mice, which had increased total fat mass and increased mass of individual fat pads (Supplementary Fig. 1a, b). The expression of *App* was increased in adipose tissue of obese mice, but not in the liver or skeletal muscle (Fig. 1a). Similarly, *Bace1* (Fig. 1b) and *Psen1* (Fig. 1c), which encodes the presenilin 1 subunit of γ-secretase, were also increased in adipose tissue of obese mice, but not in the liver or skeletal muscle. The activity of BACE1 was increased in adipose tissue and reduced in the liver of obese mice (Fig. 1d). Adipose tissue explants were found to release both Aβ_40_ and Aβ_42_ but this was not different between control and obese mice when expressed relative to explant mass (Fig. 1e). However, when accounting for total fat pad mass, adipose tissue of obese mice released more Aβ_40_ and Aβ_42_ (Fig. 1f). Release of Aβ_42_ was inhibited by incubation of explants with Brefeldin A (BFA; Supplementary Fig. 1c), an inhibitor of exocytosis^20^, indicating that adipose tissue actively secretes Aβ isoforms. The plasma concentration of Aβ_42_, but not Aβ_40_, was elevated in obese mice (Fig. 1g). Similar to findings in humans^21^, plasma Aβ_42_ was significantly correlated with fat mass (Supplementary Fig. 1d) but not with lean mass (Supplementary Fig. 1e). Together these data show that Aβ isoforms are released from adipose tissue, which is associated with elevated circulating Aβ_42_ in obesity.

### Aβ_42_ administration reprograms cardiac glucose metabolism

**Fig. 2:**
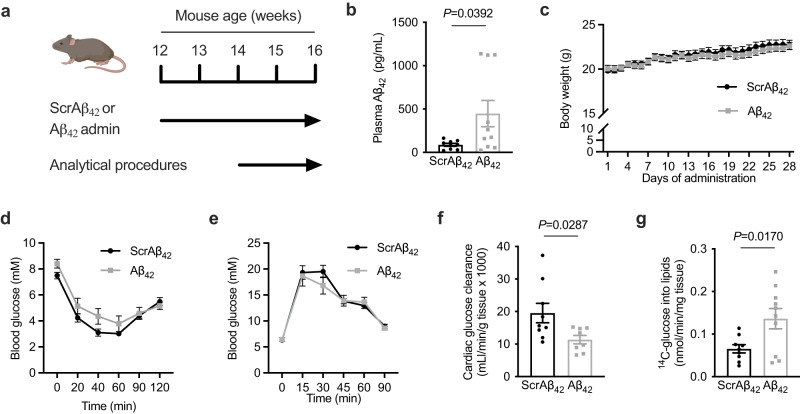
Aβ_42_ administration reprograms cardiac metabolism. **a** schematic of experiment where mice were administered Aβ_42_ or scrambled Aβ_42_ (ScrAβ_42_; 1 μg/day i.p.) for 4 weeks and analytical procedures were performed in final two weeks. **b** plasma Αβ_42_ in mice 5 h after administration of ScrAβ_42_ (*n* = 9) or Aβ_42_ (*n* = 10; unpaired t-test). **c** body weight in mice administered ScrAβ_42_ or Aβ_42_. (*n* = 10/group). **d** blood glucose during an insulin tolerance test in mice administered ScrAβ_42_ or Aβ_42_ (*n* = 10/group). **e**, blood glucose during a glucose tolerance test in mice administered ScrAβ_42_ or Aβ_42_ (*n* = 10/group). **f** cardiac glucose clearance in mice administered ScrAβ_42_ (*n* = 10) or Aβ_42_ (*n* = 9; unpaired t-test). **g** ^14^C-glucose incorporation into lipids in mice administered ScrAβ_42_ (*n* = 9) or Aβ_42_ (*n* = 10; unpaired t-test). All data are mean ± SEM. Statistical tests are two-tailed. Source data are provided in the Source Data file. Elements of **a** are created with BioRender.com.

To examine the effect of increased circulating Aβ_42_ on systemic metabolism, mice were administered Aβ_42_ or a peptide corresponding to a scrambled Aβ_42_ sequence (ScrAβ_42_; 1μg/day i.p.) for 4 weeks (Fig. 2a). Recombinant human Aβ_42_ was used as it has a greater propensity to aggregate than the mouse sequence and is more likely to reveal any pathological effects of raised Aβ_42_ concentrations^22^. These Aβ_42_ peptides appeared as monomers and low molecular weight aggregates by SDS-PAGE (Supplementary Fig. 2a). This administration regimen increased plasma Aβ_42_ ~ 4-fold (Fig. 2b) but had no effect on body weight (Fig. 2c) or composition (Supplementary Fig. 2b, c). Similarly, Aβ_42_ administration had no effect on blood glucose during both insulin (Fig. 2d) and glucose tolerance tests (GTT; Fig. 2e), or on glucose-stimulated insulin secretion (Supplementary Fig. 2d). However, further analysis of glucose fate throughout the GTT using both 2-^2^H-deoxyglucose and 1-^14^C-glucose tracers revealed that glucose clearance by the heart was reduced in mice administered Aβ_42_ (Fig. 2f). In contrast, there were no differences in glucose clearance by skeletal muscle or adipose tissue (Supplementary Fig. 2e). Furthermore, in mice administered Aβ_42_ glucose incorporation into lipids was increased (Fig. 2g), which was associated with a trend (*p* = 0.0830) towards increased cardiac triglycerides (TG; Supplementary Fig. 2f). These alterations in cardiac metabolism in mice administered Aβ_42_ were independent of changes in plasma free fatty acids and lipids (Supplementary Fig. 2g–j). These data show that increasing systemic Aβ_42_ alters cardiac glucose metabolism.

### Aβ_42_ administration induces cardiac dysfunction

**Fig. 3:**
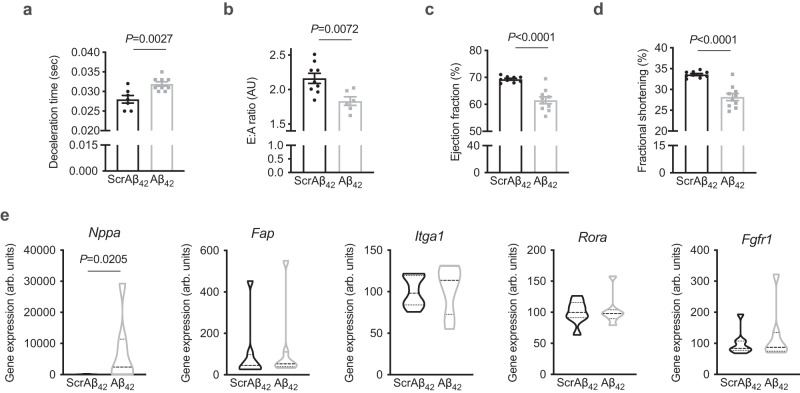
Aβ_42_ administration induces cardiac dysfunction. **a** deceleration time in mice administered ScrAβ_42_ or Aβ_42_ (unpaired t-test; *n* = 7 and 9/group respectively). **b** E:A ratio in mice administered ScrAβ_42_ or Aβ_42_ (unpaired t-test; *n* = 9 and 6/group respectively). **c** ejection fraction in mice administered ScrAβ_42_ or Aβ_42_ (unpaired t-test; *n* = 9 and 10/group respectively). **d** fractional shortening in mice administered ScrAβ_42_ or Aβ_42_ (unpaired t-test; *n* = 9 and 10/group respectively). **e** Expression of *Nppa* (Mann-Whitney test, U = 8), *Fap, Itga1, Rora* and *Fgfr1* in hearts of mice administered ScrAβ_42_ or Aβ_42_ (*n* = 8 and 7/group respectively). All data are mean ± SEM. Statistical tests are two-tailed. Source data are provided in the Source Data file.

Obesity results in reprogramming of cardiac metabolism that includes impaired glucose uptake and oxidation for a given insulin concentration^23^. These alterations in cardiac metabolism have been linked to impairments in cardiac relaxation and left ventricular (LV) filling^24^. Although increased filling pressures can overcome this diastolic dysfunction in early phases of the disease^25^, longer-term consequences include LV hypertrophy and eventual heart failure^25^. This form of heart failure most commonly manifests as heart failure with preserved ejection fraction (HFpEF)^25,26^. Hence, obesity-induced alteration of cardiac metabolism is a precipitating event leading to obesity-induced heart failure^24,25^. Given the effect of Aβ_42_ on cardiac metabolism, the effect of Aβ_42_ on cardiac function was examined by echocardiography (Supplementary Fig. 3a). Administration of Aβ_42_ had wide-ranging effects on cardiac function, including impaired diastolic function, represented by increased deceleration time (Fig. 3a) and reduced E:A ratio (Fig. 3b), indicating impairments in cardiac relaxation. Furthermore, mice administered Aβ_42_ displayed evidence of impaired systolic function, including reduced ejection fraction (Fig. 3c) and fractional shortening (Fig. 3d). Mice administered Aβ_42_ had largely normal cardiac morphology when compared with mice administered ScrAβ_42_ (Supplementary Table 1). To better understand how Aβ_42_ impairs cardiac function, profiling of genes that are differentially expressed in human heart failure specifically in the major cell types of the heart^27^ was performed. In hearts of mice administered Aβ_42_, there was a significant increase in the expression of *Nppa* (Fig. 3e), which encodes natriuretic peptide A, and is increased in cardiomyocytes in human heart failure^27^. In contrast, there were no changes in the expression of *Fap, Itga1, Rora* and *Fgfr1*, which are increased in heart failure in cardiac fibroblasts, pericytes, smooth myocytes and endothelial cells, respectively^27^. Together, these data indicate that Aβ_42_ impairs cardiac function, likely through effects on cardiomyocytes.

In the context of AD, Aβ_42_ is considered the most pathogenic of the Aβ peptides because of its propensity to aggregate^28^. However, Aβ_40_ is also found in plasma^17,29^. To determine whether Aβ_40_ also has deleterious effects on the heart, mice were administered Aβ_40_ (1μg/day i.p.) for 4 weeks followed by echocardiographic assessment of cardiac function and morphology, as was performed for Aβ_42_. Administration of Aβ_40_ markedly increased the circulating concentration of Aβ_40_ (Supplementary Fig. 3b) but had no effect on body weight and composition (Supplementary Fig. 3c–e), and had no effect on any index of cardiac function or morphology (Supplementary Table 2 and Supplementary Fig. 3f). Together with our previous findings, these data suggest that circulating Aβ_42_, but not Aβ_40_, has deleterious effects on the heart.

### An Aβ neutralising antibody prevents obesity-induced impairment of cardiac relaxation

**Fig. 4:**
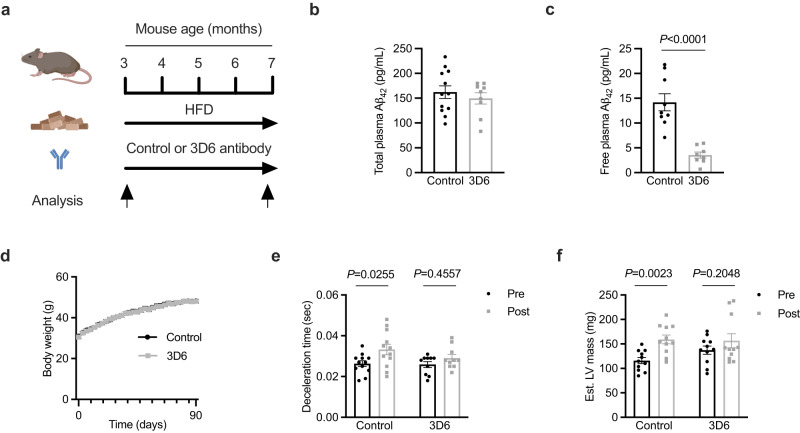
An Aβ_42_ neutralising antibody prevents obesity-induced impairment of cardiac relaxation. **a** schematic of experiment where mice fed a high fat diet (HFD) for 4 months and were simultaneously administered a control antibody or an Aβ_42_ neutralising antibody (3D6) once weekly. Analysis of cardiac function and morphology was performed at the beginning and end of the experiment. **b** total (*n* = 12 and 9/group respectively), and; **c**, free (*n* = 9 and 8/group respectively) Aβ_42_ in plasma of mice administered control or 3D6 antibodies (unpaired t-test). **d** body weight of mice administered control or 3D6 antibodies (*n* = 12/group). **e** deceleration time in mice prior to and after 4 months of high-fat feeding and antibody administration (two-way repeated measures ANOVA (time *P* = 0.0140, *F*(1,20) = 7.257) and Sidak’s multiple comparisons test *P*.adjusted; *n* = 12 and 10/group respectively). **f** estimated left ventricle (LV) mass in mice prior to and after 4 months of high-fat feeding and antibody administration (two-way repeated measures ANOVA (time *P* = 0.0010, *F*(1,20) = 14.93) and Sidak’s multiple comparisons test *P*.adjusted; *n* = 11/group). All data are mean ± SEM. Statistical tests are two-tailed. Source data are provided in the Source Data file. Elements of **a** are created with BioRender.com.

Having established that Aβ_42_ release from adipose tissue is increased in obesity and that raising systemic concentrations of Aβ_42_ negatively impacts cardiac metabolism and function, we next sought to determine whether Aβ_42_ impairs cardiac relaxation in obesity, which is the initial defect in cardiac function that ultimately leads to heart failure^30^. We and others have previously established that deletion of *App* or components of the APP proteolytic machinery in mice increases energy expenditure and confers resistance to obesity^31,32^. Hence, these genetic models cannot be used to assess the role of Aβ_42_ on cardiac function in obesity. Instead, the mouse-specific Aβ neutralising antibody 3D6 was employed to address this question. The binding of 3D6 to Aβ_42_ prevents Aβ_42_ interacting with receptors and the cellular internalisation of Aβ_42_^33^. The humanised version of 3D6, bapineuzumab, reached phase III trials for AD^34^. Although it had excellent target engagement and an acceptable safety profile, it failed to produce meaningful improvements in cognition in AD patients^34^. Cardiovascular conditions were exclusion criteria for this and other AD trials, meaning that data on any potential cardiovascular effects are not available. Therefore, to determine whether Aβ_42_ mediates the adverse effects of obesity on cardiac function, mice were fed a high-fat diet and were simultaneously administered 3D6 or a control antibody (0.75 mg/kg, i.p. once weekly) for a period of 4 months. Cardiac function and morphology were assessed by echocardiography at the beginning and conclusion of the experiment (Fig. 4a, Supplementary Fig. 4a). The initiating event in the development of obesity-induced cardiac dysfunction and eventual heart failure is impaired cardiac relaxation, which is characterised by increased deceleration time^35–37^. We have previously observed impairments in deceleration time in this model between 3-4 months of high-fat feeding^38^. Consistent with previous studies^39^, 3D6 administration did not reduce total plasma Aβ_42_ concentration (Fig. 4b) but did reduce the plasma concentration of free Aβ_42_ that was unbound by antibodies (Fig. 4c). Administration of 3D6 had no effect on body weight (Fig. 4d), body composition or systemic metabolism (Supplementary Fig. 4b–f). In mice administered control antibody, deceleration time increased over the course of the high-fat feeding period (Fig. 4e). In contrast, deceleration time was not increased in mice administered 3D6 (Fig. 4e). The same result was observed when deceleration time was normalised as a percentage of the cardiac cycle (Supplementary Fig. 4g). Although initial estimated LV mass was not matched (but was not statistically different) between groups, LV mass was increased throughout the study in mice administered control antibody but was unchanged in mice administered 3D6 (Fig. 4f). A similar finding in left ventricular posterior wall thickness at diastole (LVPWd; Supplementary Table 3), an alternate index of LV mass^40^, was observed. These data suggest that systemic Aβ_42_ contributes to the obesity-induced impairment in cardiac relaxation and increase in LV mass.

### An Aβ neutralising antibody prevents further impairment of cardiac relaxation in established obesity

**Fig. 5:**
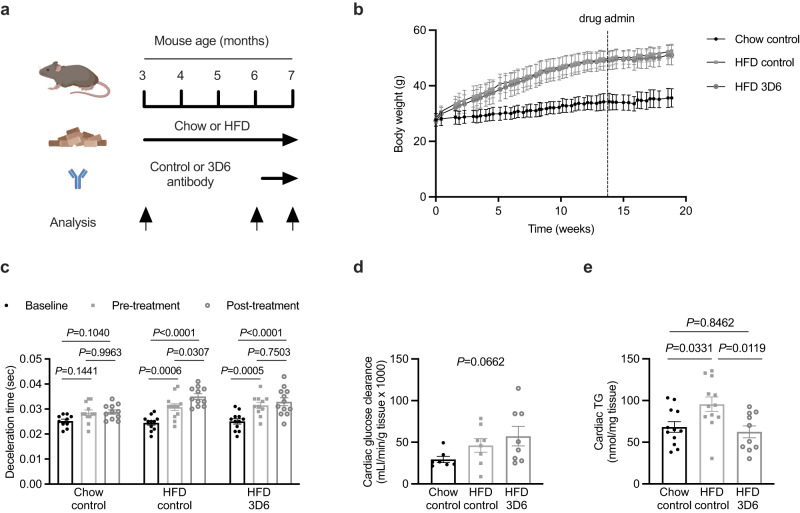
An Aβ_42_ neutralising antibody prevents further impairment of cardiac relaxation in established obesity. **a** schematic of experiment where mice fed either chow or a high-fat diet (HFD) for 4 months and were administered a control antibody or an Aβ_42_ neutralising antibody (3D6) once weekly in the final month of the diet period. Analysis of cardiac function and morphology was performed at the beginning and of the experiment (baseline), prior to the treatment period (pre-treatment) and at the end of the treatment period (post-treatment). **b** body weight over time in mice fed regular chow or HFD and administered control or 3D6 antibodies (*n* = 12/group). **c** deceleration time in mice at Baseline, pre-treatment and post-treatment after 4 months of chow or high fat feeding and antibody administration (mixed-effects model (time *P* < 0.0001, *F*(2,93) = 31.56; treatment *P* = 0.0151, *F*(2,93) = 4.390) and Sidak’s multiple comparisons test *P*.adjusted; *n* = 12/group). **d** cardiac glucose clearance (*n* = 8, 9 and 9/group respectively), and; **e** cardiac triglycerides (TG; One-way ANOVA, *P* = 0.0082, F(2,31) = 5.624; *n* = 12, 12 and 10/group respectively) in mice fed regular chow and administered control antibody, or mice fed a high fat diet (HFD) and administered control or 3D6 antibodies. All data are mean ± SEM. Statistical tests are two-tailed. Source data are provided in the Source Data file. Elements of **a** are created with BioRender.com.

The finding that Aβ_42_ antagonism prevents obesity-induced impairment of cardiac relaxation raises the possibility that targeting Aβ_42_ could be an effective treatment for established obesity-induced cardiac dysfunction. To test this hypothesis, mice were fed either standard chow or a high fat diet for 4 months and were administered 3D6 or control antibodies for the final 4 weeks of the experiment. Echocardiography to assess cardiac function and morphology was performed at the beginning of the experiment (baseline), prior to antibody treatment (pre-treatment) and at the conclusion of the study (post-treatment; Fig. 5a, Supplementary Fig 5a). Mice fed a high-fat diet had increased body weight (Fig. 5b), fat mass and fasting plasma insulin compared with chow-fed mice; however, 3D6 administration had no effect on these parameters (Supplementary Fig. 5b–e). There was no change in deceleration time in chow fed mice administered control antibody throughout the experiment (Fig. 5c). Mice fed high fat diet and administered control antibody had an increase in deceleration time from baseline to pre-treatment, which increased further from pre-treatment to post-treatment (Fig. 5c). Mice fed high fat diet and administered 3D6 antibody had an increase in deceleration time from baseline to pre-treatment, however unlike mice administered control antibody, there was no further increase in deceleration time from pre-treatment to post-treatment (Fig. 5c). These findings were consistent when deceleration time was expressed as a percentage of the cardiac cycle (Supplementary Fig. 5f). Short term administration of 3D6 in established obesity did not alter any index of LV mass (Supplementary Table 4) but tended to increase cardiac glucose clearance (Fig. 5d and Supplementary Fig. 5g) and normalised cardiac TG concentrations (Fig. 5e). These data suggest that Aβ_42_ antagonism halts further deterioration of cardiac relaxation in established obesity.

### Aβ_42_ reduces mitochondrial transcriptional programs and accumulates in cardiac mitochondria in obesity

**Fig. 6:**
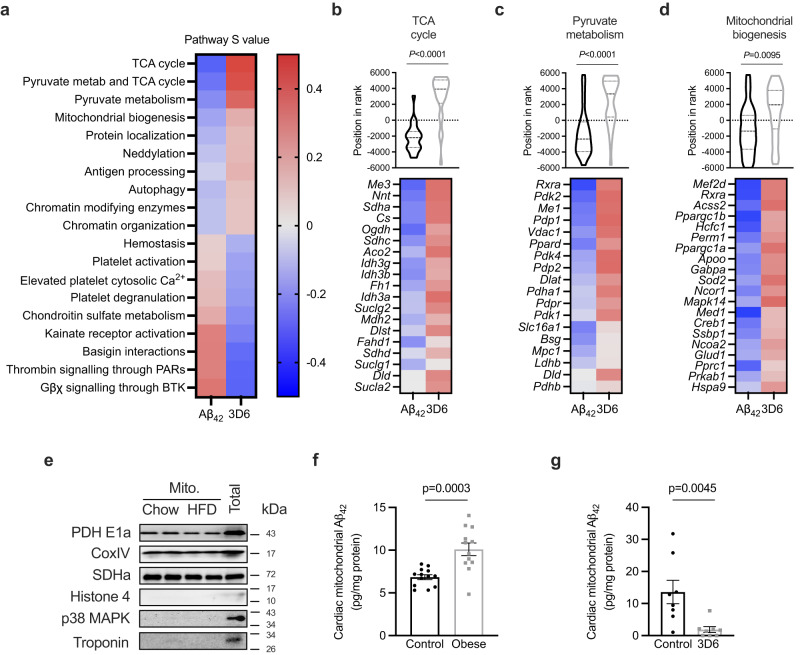
Aβ_42_ impairs mitochondrial transcriptional programs and accumulates in cardiac mitochondria in obesity. **a** heat map of Reactome pathways reciprocally regulated in the hearts of mice from Aβ_42_ (Fig. 2a) and 3D6 (Fig. 3a) administration studies, determined by MITCH analysis from bulk RNA-seq data. **b** TCA cycle pathway rank (*P*.adjusted MANOVA test) and heat map of TCA cycle genes in the hearts of mice from Aβ_42_ and 3D6 administration studies. **c** pyruvate metabolism pathway rank (*P*.adjusted MANOVA test) and heat map of pyruvate metabolism genes in the hearts of mice from Aβ_42_ and 3D6 administration studies. **d** mitochondrial biogenesis pathway rank (*P*.adjusted MANOVA test) and heat map of mitochondrial biogenesis genes in the hearts of mice from Aβ_42_ and 3D6 administration studies. **e** characterisation of enriched mitochondrial fractions from hearts of chow or high fat diet (HFD)-fed mice. **f** Aβ_42_ in mitochondrial fractions isolated from hearts of chow or HFD-fed mice (unpaired t-test; *n* = 13 and 12/group respectively). **g** Aβ_42_ in mitochondrial fractions isolated from hearts of mice fed HFD and administered control or 3D6 antibodies (Fig. 3a; unpaired t-test; *n* = 8/group). All data are mean ± SEM. Statistical tests are two-tailed. Source data are provided in the Source Data file.

In the context of AD, Aβ_42_ has highly complex cellular effects through a myriad of mechanisms depending on the target cell type and its aggregation state^41^. To gain unbiased insights into the deleterious effects that Aβ_42_ has on the heart, a multidimensional transcriptomic approach was employed. The transcriptomic profile of hearts obtained in Aβ_42_ administration studies (Fig. 2a) were compared with the profile of hearts obtained in the 3D6 administration study (Fig. 4a) and pathways reciprocally regulated were identified using MITCH^42^. It was reasoned that reciprocally regulated pathways from these experiments represent geneset changes that are fundamental to the effects of Aβ_42_ on the heart. Global gene expression across experiments was evenly distributed (Supplementary Fig. 6a and b). Multi-contrast enrichment analysis identified the TCA cycle, pyruvate metabolism and mitochondrial biogenesis pathways with the largest effect sizes that were reduced by Aβ_42_ administration and increased by 3D6 administration (Fig. 6a and Supplementary Table 5). These pathways are dysregulated in obesity-induced cardiac dysfunction and have been linked with disrupted energetics that impair cardiac relaxation^24,43–45^. The transcriptional reprogramming of the TCA cycle included altered regulation of genes encoding TCA cycle enzymes (Fig. 6b), while transcriptional regulation of pyruvate metabolism included subunits of pyruvate dehydrogenase (PDH) and PDH kinases (PDKs; Fig. 6c). Regulated genes in the mitochondrial biogenesis pathway were mainly of nuclear transcription factors controlling the mitochondrial biogenesis process (Fig. 6d). Interestingly, several receptor pathways were increased by Aβ_42_ administration and reduced by 3D6 administration (Fig. 6a and Supplementary Table 5). The widespread transcriptional reprogramming of mitochondrial pathways likely indicates a secondary response to the effects of Aβ_42_ on mitochondria. Aβ_42_ can be taken up by mitochondria, where it can impact numerous aspects of mitochondrial biology^15,46,47^. To examine whether Aβ_42_ accumulates in mitochondria of the heart in obesity, enriched mitochondrial fractions were isolated from control and obese mice (Fig. 6e). Cardiac mitochondria from obese mice were found to have elevated concentrations of Aβ_42_ (Fig. 6f). Interestingly, mitochondrial Aβ_42_ was also increased in the skeletal muscle of obese mice, but not in adipose tissue or in the liver (Supplementary Fig. 6c), suggesting that striated muscle specifically has a propensity to accumulate Aβ_42_ in obesity. Furthermore, Aβ_42_ concentrations were reduced in cardiac mitochondria in obese mice that were administered 3D6 antibodies (Fig.6g), consistent with reports that this antibody enhances tissue Aβ clearance^48^.

### Aβ_42_ inhibits mitochondrial complex I in cardiomyocytes

**Fig. 7:**
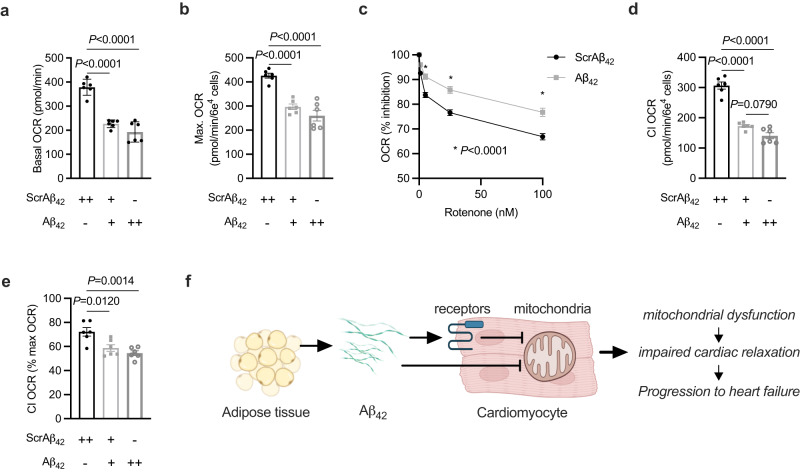
Aβ_42_ inhibits mitochondrial complex I in cardiomyocytes. **a** Basal oxygen consumption rate (OCR) in primary mouse neonatal cardiomyocytes (NVCM) exposed to ScrAβ_42_ or Aβ_42_ (ScrAβ_42_ at 300 (++), 100 (+) and 0pM (−) and co-incubated with Aβ_42_ at 0 (−), 200 (+) and 300pM (++) for 48 hrs (one-way ANOVA (*P* < 0.0001; F(2,15) = 53.5) with Sidak’s repeated measures test *P*.adjusted; *n* = 6 biological replicates/group). **b** maximal OCR in primary NVCM exposed to ScrAβ_42_ or Aβ_42_ for 48 hrs (one-way ANOVA (*P* = 0.0001; F(2,15) = 31.2) with Sidak’s repeated measures test *P*.adjusted; *n* = 6 biological replicates/group). **c** inhibition of respiration in response to increasing concentrations of rotenone in primary NVCM exposed to ScrAβ_42_ or Aβ_42_ for 48 hrs (multiple t-tests *P*.adjusted; *n* = 5 biological replicates/group). **d** absolute complex I (CI) OCR in primary NVCM exposed to ScrAβ_42_ or Aβ_42_ for 48 hrs (one-way ANOVA (*P* < 0.0001; F(2,15) = 88.4) with Sidak’s repeated measures test *P*.adjusted; *n* = 6 biological replicates/group). **e** complex I (CI) OCR as a percentage of total respiratory capacity in primary NVCM exposed to ScrAβ_42_ or Aβ_42_ for 48 hrs (one-way ANOVA (*P* < 0.0012; F(2,15) = 10.8) with Sidak’s repeated measures test *P*.adjusted; *n* = 6 biological replicates/group). All data are mean ± SEM. All statistical tests are two-tailed. Source data are provided in the Source Data file. **f** schematic of proposed model whereby adipose tissue release of Aβ_42_ is increased in obesity resulting in higher circulating levels of Aβ_42_. Increased circulating Aβ_42_ inhibits cardiomyocyte mitochondrial ATP production and causes diastolic dysfunction, which starts the progression towards heart failure. The effects of Aβ_42_ on cardiomyocytes could be mediated through receptor-mediated signalling, receptor-mediated internalisation, or direct internalisation. **e** was created with BioRender.com.

Identification of the mitochondrial target of Aβ_42_ was further explored. Upon cellular uptake and mitochondrial import, Aβ_42_ is known to physically interact with and inhibit complex I of the respiratory chain^49^. Complex I is a candidate target for Aβ_42_ in the context of obesity, which is characterised by reduced complex I function in the heart^43,50,51^. As gene expression profiling suggested that the effects of Aβ_42_ on mitochondria were specific for cardiomyocytes (Fig. 2l), a primary neonatal ventricular cardiomyocyte model system was used to examine whether Aβ_42_ inhibits complex I in cardiomyocytes. Exposure of cardiomyocytes to increasing concentrations of Aβ_42_ for 48hrs reduced cardiomyocyte oxygen consumption rate (OCR; Fig. 7a) as well as maximal respiratory capacity (Fig. 7b). This was not explained by a loss of cell viability (Supplementary Fig. 7a) and indicate that Aβ_42_ has direct effects on cardiomyocytes. Exposure of Aβ_42_ to FAO hepatocytes did not have any effect on basal respiration (Supplementary Fig. 7b), suggesting cell type-specific effects of Aβ_42_. Sensitivity to the complex I inhibitor rotenone (Fig. 7c), but not the unrelated ATP synthase inhibitor oligomycin (Supplementary Fig 7c), was altered following exposure of cardiomyocytes to Aβ_42_ and was associated with reduced complex I-mediated respiration (Fig. 7d). To dissect whether the effects of Aβ_42_ on complex I could be explained by the reduction in respiratory capacity, complex I-mediated respiration was expressed relative to maximal respiratory capacity and was reduced by exposure to Aβ_42_ (Fig. 7e). This provides further evidence that Aβ_42_ inhibits complex I of the respiratory chain as well as reducing mitochondrial capacity in cardiomyocytes, both of which have been observed in heart failure^43,51–53^. These findings suggest that systemic Aβ_42_ is internalised and taken up by mitochondria in cardiomyocytes, where it can impair mitochondrial respiratory function.

## Discussion

Our findings have revealed an unexpected role for Aβ_42_ in the aetiology of obesity-induced cardiac dysfunction (Fig. 7f). Factors such as increased adiposity, myocardial lipid accumulation and cardiomyocyte mitochondrial dysfunction have all been implicated in the development of diastolic dysfunction in obesity. Data generated in the present study indicates that Aβ_42_ is a unifying link between all of these factors and is supported by a recent study, published while the current manuscript was in preparation, proving the association between circulating Aβ and heart failure in the general population^54^. Our data advances our understanding of the impact of obesity on the heart and identify an actionable target for therapeutic intervention in obesity-induced heart failure.

In humans, both total fat mass and visceral fat mass are associated with impaired myocardial energetics and diastolic dysfunction^24^. Although increased free fatty acid availability that leads to myocardial lipid accumulation is one potential mechanism describing this association, diastolic dysfunction in humans occurs with moderate visceral adiposity before the appearance of myocardial lipid accumulation^24^. One interpretation of these data is that factors released by adipose tissue other than fatty acids, such as Aβ_42_, are more important contributors to obesity-induced diastolic dysfunction. While adipose tissue could be a major source of Aβ_42_ in obesity, the exact cell types involved remain obscure. In addition to adipocytes, vascular endothelial cells and immune cells, which infiltrate adipose tissue in obesity, are all candidate cell types that express high levels of APP and the APP processing machinery and could release Aβ peptides, particularly under metabolic conditions associated with obesity^55–57^.

Increasing systemic Aβ_42_ reprogrammed cardiac glucose metabolism, including reduced glucose clearance and increasing glucose incorporation into lipids. It is well established that impairments in cardiac glucose metabolism are associated with impairments in cardiac function^58–61^. Consistent with these findings, administration of Aβ_42_ to normal mice resulted in wide-ranging defects in both diastolic and systolic function. However, the early stages of obesity, in both mice and humans, are generally not associated with systolic dysfunction, despite elevated circulating Aβ_42_. This discrepancy is likely due to whole-body metabolic and hormonal alterations in obesity that minimise the impact of Aβ_42_ on the heart, restricting its effects to relaxation defects that manifest as diastolic dysfunction.

While preparing this manuscript, it was reported that plasma concentrations of both Aβ_42_ and Aβ_40_ are associated with heart failure in the general population, particularly in males^54^. While the association was stronger for Aβ_40_, this could be due to the greater propensity for Aβ_42_ to leave the circulation and accumulate in vascular beds and in tissues^62^. Indeed, in our administration studies, the delivery of equal amounts of Aβ_40_ and Aβ_42_ resulted in a greater plasma excursion of Aβ_40_ (Fig. 2b and Supplementary Fig. 3b). Our findings are also consistent with associative observations in patients with AD, whereby Aβ_42_ accumulates in the heart and is associated with diastolic dysfunction^63^.

The accumulation of Aβ_42_ in cardiac mitochondria and the Aβ_42_-mediated reduction in complex I-associated respiration in cardiomyocytes phenocopies aspects of Aβ_42_ biology in AD. A recent study suggests that other aspects of mitochondrial function are also impacted by Aβ_42_ in cardiomyocytes^64^. The extent to which the effects of Aβ_42_ on complex I account for the metabolic and contractile impairments that occur in cardiomyocytes in obesity in vivo remains to be determined. However, we hypothesise that complex I is a major site of metabolic impairment in the heart in obesity and could explain the defects in glucose oxidation that are observed in heart failure. Complete oxidation of glucose primarily generates NADH as the intermediate electron carrier, which is oxidise by complex I of the respiratory chain. In contrast, beta-oxidation of fatty acids generates substantial FADH_2_ that is oxidised by complex II of the respiratory chain and complex I inhibition increases fatty acid oxidation in a variety of cell types^65–67^. A limitation of the current study is that the effect of Aβ_42_ on mitochondria was examined in neonatal cardiomyocytes. This model was used rather than adult cardiomyocytes, as the latter are prone to extensive cell death and alterations in cellular identity in the first 24-48 hr of culturing^68^, which could confound the effects of Aβ_42_. It is well established that neonatal cardiomyocytes oxidise proportionally more glucose and less fatty acids than adult cardiomyocytes^69^. Therefore, further studies examining how Aβ_42_ impacts mitochondrial substrate utilisation are warranted. As adult primary cardiomyocytes quickly de-differentiate and reprogram mitochondrial capacity accordingly^70^, sophisticated studies in vivo will be required to properly dissect the metabolic effects of Aβ_42_ on cardiomyocytes and the heart.

This study supports the idea that therapies originally designed for AD that reduce or antagonise Aβ_42_ could be repurposed to treat and/or prevent obesity-induced heart failure^71^, particularly those with HFpEF, for which treatment options are limited^25^. This is an urgent unmet need as this patient group has a 5-year mortality rate of up to 75% and currently affects up to 8% of people in certain populations^30^. Furthermore, the prevalence of HFpEF is predicted to rise in parallel with rising rates of obesity^30^. A suite of biologics and small molecules has been developed for AD that antagonise or reduce Aβ_42_, many with well-established safety and tolerability profiles in humans. These diverse therapies with distinct mechanisms of action could therefore be rapidly assessed as new treatments for heart failure in obesity.

In conclusion, this study has identified a link between adiposity, Aβ_42_ release from adipose tissue and impairments in cardiac metabolism and function. The data generated highlight mechanistic similarities in the development of obesity-induced cardiac dysfunction and AD and support the idea that repurposing drugs originally developed for AD could be an approach to treat and manage heart failure.

## Methods

### Animal studies

Male C57BL6J mice were used for all mouse experiments and were obtained from the Animal Resources Centre (Perth, WA, Australia) and were group housed with four to five mice per cage with a 12 h light/dark cycle at 22 °C with humidity 20–60%. Animals had ad libitum access to food (12.8 kJ/g, 6% fat, 20% protein; Barastoc Mouse Maintenance Cube, Ridley AgriProducts) and water. All procedures involving animals were approved by The Deakin University Animal Welfare Committee (A58-2010, G07-2013, G15-2017 and G08-2020), which is subject to the Australian Code for the Responsible Conduct of Research.

#### High-fat feeding

At 12 weeks of age, mice were randomly assigned to regular chow or high-fat diet (HFD; 43% digestible energy from fat, 20% from sucrose; SF04-001 Specialty Feeds). Throughout the diet period, mice were weighed weekly. After three months of the diet period, mice underwent body composition assessment using EchoMRI. Two days later, mice were fasted for 5 hrs before being humanely killed by cervical dislocation. Epididymal and inguinal adipose tissues were carefully excised and weighed and were either immediately frozen and stored at −80 °C for later analysis or were used for ex vivo adipose explant experiments.

#### Aβ peptide administration

Lyophilised recombinant Aβ_42_ (#AG912, Millipore) and scrambled control peptide (ScrAβ_42_; #AG916, Millipore) were resuspended in 1% NH_4_OH and aliquoted at 200 ng/ml in H_2_O and stored at −80 °C for no longer than 4 weeks. Peptide administration began when mice were 10-12 weeks of age and involved a daily i.p. injection of 1μg of peptide. Throughout the peptide administration period, body weight was measured daily and food intake was measured twice weekly, and body composition was measured weekly by EchoMRI. After two weeks, plasma Aβ_42_ was determined from samples collected 5 hr after recombinant ScrAβ_42_ or Aβ_42_ administration. After 3 weeks, an insulin tolerance test (ITT; i.p. 0.75U/kg) was performed following a 5 hr fast and 5 hr after peptide administration. Blood glucose was measured at baseline and after 20, 40, 60, 90 and 120 min after glucose administration. One week later, a glucose tolerance test (GTT; i.p 2 g/kg with 5μCi ^3^H-2-deoxyglucose and 5μCi ^14^C-glucose) was performed after a 5 hr fast and 5 hr after peptide administration. Blood glucose was measured at baseline and after 15, 30, 45, 60 and 90 min after glucose administration. Plasma was obtained at 0, 15, 30, 60 and 90 min for insulin determination and the measurement of tracer-specific activity. Mice were humanely killed by cervical dislocation immediately after the GTT. Blood was collected for plasma and tissues were dissected, rapidly frozen and stored at −80 °C for later analysis. Another cohort of mice were administered ScrAβ_42_ or Aβ_42_ for four weeks, as described above. After four weeks of peptide administration, mice were anaesthetised by isoflurane inhalation before undergoing echocardiography. Two days later, mice were humanely killed by cervical dislocation and blood was obtained for plasma and tissues were dissected, rapidly frozen and stored at −80 °C for later analysis. Another cohort of mice were administered ScrAβ_40_ or Aβ_40_ (Millipore) for four weeks, as described above. After four weeks of peptide administration, mice were anaesthetised by isoflurane inhalation before undergoing echocardiography. Two days later, mice were humanely killed by cervical dislocation and blood was obtained for plasma and tissues were dissected, rapidly frozen and stored at −80 °C for later analysis.

#### 3D6 administration

For the 3D6 prevention study, 12-week-old mice were assessed for baseline cardiac structure and function by echocardiography and were assigned to one of two groups such that deceleration time was matched. One week later, mice were fed HFD (as described above) and then received control antibody (IgG2a isotype antibody, #BE-0085 BioXCell) or 3D6 antibody (#TAB-0809CLV, Creative Biolabs) weekly via i.p. injection (0.75 mg/kg). Body weight was measured twice per week, while body composition was measured every two weeks throughout the study. After 10 weeks of high-fat feeding, mice underwent an ITT and then a GTT after 11 weeks of high-fat feeding. After 12 weeks of high-fat feeding mice underwent echocardiography and two weeks later were humanely killed by cervical dislocation following a 5 hr fast. Blood was collected for plasma and tissues were dissected, rapidly frozen and stored at −80 °C for later analysis. In the 3D6 reversal study, 12-week-old mice were assessed for baseline cardiac structure and function by echocardiography and were assigned to one of three groups such that deceleration time was matched. One week later, one group of mice remained on standard chow, while the other two groups were fed a high-fat diet. Body weight was measured twice per week throughout the study. After 13 weeks of high-fat feeding, mice underwent echocardiography for pre-treatment assessment of cardiac function and morphology. Mice were then administered control antibody or 3D6 antibody weekly via i.p. injection (0.75 mg/kg) for the remainder of the study. Four weeks later, mice underwent post-treatment assessment of cardiac function and morphology, followed by assessment of body composition by EchoMRI two days later. One week later, mice were humanely killed by cervical dislocation following a 5 h fast. Blood was collected for plasma and tissues were dissected, rapidly frozen and stored at −80 °C for later analysis.

#### Echocardiography

Echocardiography was performed using a Phillips HD15 diagnostic ultrasound system with 15 MHz linear-array transducer^38,72,73^. In line with published recommendations^74^, depth of anaesthesia was modulated to maintain heart rate between 400 and 650 bpm. Images were not quantified when heart rate was ≤ 350 bpm. Transmitral Doppler imaging was used to assess LV filling velocity and deceleration time was determined from the slope of the E-wave. Only E-waves where there was sufficient separation from the A-wave to allow slope determination were quantified. Aortic valve Doppler imaging was used to assess aortic flow and ejection times. Ejection fraction and fractional shortening (FS) were derived from M-Mode measurements as indicators of LV systolic and contractile function and chamber morphology was also determined from M-mode measures. Echo images were analysed using the HD15 system in a blinded manner. The average value of each index from 2-4 cardiac cycles was used.

### Adipose tissue explants

Prior to the ex vivo incubation protocol, all consumables such as 12-well plates, tips, and tubes were blocked in 5% bovine serum albumin (BSA) to minimise binding of Aβ_42_ to plasticware^75^. Tests with recombinant Aβ_42_ revealed recovery of ~95% of Aβ_42_ using this method. Upon collection of inguinal adipose tissue samples, four ~10–15 mg tissue portions were isolated and each of them was placed in a well with 500 uL of DMEM media supplemented with 10% BSA. Three samples were incubated in vehicle (0.1% DMSO), while one incubated in 10μM Brefeldin A. Each tissue was individually weighed, the starting time of the incubation recorded, and the plate was placed at 37 °C in 10% CO_2_ for 24 h. Following the 24 h period, both media and the tissue were rapidly collected, and subsequently snap-frozen, for further analyses. High-sensitivity ELISA kits were used to quantify Aβ_40_ (#294-62501 human/rat/mouse, FUJIFILM Wako) and Aβ_42_ (#292-64501 human/rat/mouse high sensitivity, FUJIFILM Wako) and the protocol was followed as per the manufacturer’s protocol. In brief, samples were brought to room temperature, vortexed, and 100 μl of sample was placed in each well, together with the respective internal controls for each plate. Each plate was then sealed and refrigerated overnight. Following this, the solutions were discarded, and each well was washed 5 times before addition of 100 μl of HRP-conjugated antibody. Following 1 hour of incubation, the solution was discarded and the washing step was repeated, with subsequent addition of 100 μl TMB solution to initiate the HRP reaction at room temperature in the dark. Following 30 minutes, 100 μl of Stop-solution was added to terminate the reaction and the absorbance (at 450 nm) was subsequently measured to determine the protein levels.

### Glucose clearance and fate

The LV of the heart, quadriceps skeletal muscle and epididymal adipose tissue (15-25 mg) were used to determine 2-^2^H-deoxyglucose clearance as we have previously described^73^. To determine 1-^14^C-glucose incorporation into glycogen, ~10-15 mg of LV tissue was digested in 1 M KOH at 70 °C for 20 min and glycogen was precipitated with saturated Na_2_SO_4_, washed twice with 95% ethanol and resuspended in acetate buffer (0.84% sodium acetate, 0.46% acetic acid, pH 4.75) containing 0.3 mg/mL amyloglucosidase (Sigma). Glycogen was allowed to digest overnight at 37 °C before being assayed for glucose content using the glucose oxidase method^76^. Digested glycogen was also assessed for ^14^C-glucose incorporation by scintillation counting. To determine 1-^14^C glucose incorporation into lipids, 5–10 mg of LV tissue was homogenised in chloroform/methanol (2:1) and mixed overnight at room temperature. Organic and inorganic phases were separated by addition of 0.6% NaCl and the lower organic phase was collected and evaporated under N_2_ at 45 °C. The dried extract was resuspended in absolute ethanol and TG content was assayed using TG GPO-PAP reagent (Roche) and ^14^C-glucose incorporation by scintillation counting.

### Gene expression and analysis

Bulk RNA was isolated from LV tissue by homogenisation using a hand-held homogeniser in Trizol and RNA was isolated using RNeasy kits (Qiagen), according to manufacturer’s instructions. Sequencing libraries were generated from 0.5 µg of total RNA using TruSeq Stranded Total RNA preparation kit (Illumina) as per manufacturer’s instructions. Transcriptome-wide mRNA levels were measured using the NovaSeq 6000 Sequencing System (Illumina). Sequence Fastq files underwent quality trimming and mapping to the mouse reference transcriptome with Salmon. The reference transcriptome sequence was obtained from Gencode (version vM24). Counts were read into R (version 4.0.2)^77^. Differential expression analysis was performed separately for two contrasts (ScrAβ_42_ vs. Aβ_42_ and Control vs 3D6). Genes with fewer than 10 reads per sample on average were discarded. Differential analysis was performed with DESeq2 (version1.28.1). Multi-contrast enrichment analysis was performed with mitch (version 1.0.6) using default settings, which implements an ANOVA on ranks approach^42^. Human Reactome pathways were downloaded (19^th^ May 2020) and gene names were converted to mouse using Ensembl ortholog mapping. Gene sets with false discovery rate <0.05 were considered significant. Genesets that were significantly regulated in both contrasts were presented. The expression of *App, Bace1 and Psen1* were quantified in epididymal adipose tissue, quadriceps skeletal muscle and the liver of control and obese mice by real time RT-PCR using the following primer pairs (*App* Fwd CTTGCACGACTATGGCATGC, rev GTCATCCTCCTCTGCATCCG; *Bace1* Fwd GGAGCCCTTCTTTGACTCCC, rev CCCGTG TATAGCGAGTGGTC; *Psen1* Fwd TTCAAGAAAGCGTTGCCAGC, rev AGGGCTGCACA AGGTAATCC).

### Plasma and biochemical analyses

BACE-1 activity was assessed using a fluorescent assay kit (Merck), while plasma glycerol and plasma HDL-C were determined using colorimetric kits (Sigma), all according to manufacturer’s instructions. High-sensitivity ELISAs were used to measure plasma Aβ_40_ and Aβ_42_ (FUJIFILM Wako), as described above, and plasma insulin (ALPCO), according to manufacturer’s instructions. Plasma TG concentration was assayed using TG GPO-PAP reagent (Roche) and plasma NEFA was determined using a colorimetric kit (FUJIFILM Wako). To quantify free plasma Aβ_42_, 100μL of plasma was added to 150μL of 10 mM Tris pH 7.5 and incubated with protein G magnetic beads, for 2 hours at 4 °C on a rotating wheel, before being spun in a centrifuge at 5,000 g for 5 min at 4 °C. The supernatant was collected and 100 μL was used to quantify Aβ_42_ using a high-sensitivity ELISA, as described above. Protein G beads were blocked with 0.2% BSA in 10 mM Tris pH 7.5 overnight at 4 °C on a rotating wheel prior to the assay.

### Isolation of enriched mitochondrial fractions

Approximately 50 mg of left ventricular tissue was homogenised with a handheld homogeniser at low speed for two 10 sec bursts while on ice in 500μL of mitochondrial isolation buffer (70 mM sucrose, 220 mM mannitol, 5 mM KH_2_PO_4_, 5 mM MgCl_2_, 2 mM HEPES, 1 mM EGTA, 0.2% BSA). The homogenate was spun in a centrifuge at 800 g for 10 min at 4 °C. The supernatant was collected and spun in a centrifuge at 12,000 g for 10 min at 4 °C. The resulting pellet was resuspended in lysis buffer (50 mM Tris pH 7.5, 1 mM EDTA, 1 mM EGTA, 10% glycerol, 1% triton X-100, 50 mM NaF, 5 mM Na_4_P_2_O_7_, 1 mM Na_3_VO_4_, 1 mM DTT) and mitochondrial protein concentration was determined by the BCA method. Quantification of Aβ_42_ was performed from 200μg of mitochondrial protein.

### Western blotting

For western blotting, 10 µg of protein were denatured in SDS reducing buffer and incubated at 37 °C for 5 min before being subjected to SDS-PAGE^78^. Proteins were transferred onto polyvinylidine diflouride (PVDF) membranes, which were blocked in 1% bovine serum albumin in Tris-buffered saline and 0.05% Tween (TBST) for 1 hour at RT before being exposed to primary antibodies against pyruvate dehydrogenase (PDH) E1a subunit (Cell Signalling Technology #2784), cytochrome c oxidase subunit 4 (CoxIV; Cell Signalling Technology #4844), succinate dehydrogenase subunit A (SDHa; Cell Signalling Technology #11998), histone 4 (H4; Cell Signalling Technology #2592) p38 mitogen activate protein kinase (MAPK; Cell Signalling Technology #9212), troponin (Cell Signalling Technology #4002), amyloid beta (Moab2, Abcam ab126649) and α-tubulin (Sigma-Aldrich T6074). All antibodies were diluted in 1xTBST at a concentration of 1:1000. Membranes were washed in TBST and exposed to appropriate anti-species horseradish peroxidase (HRP) conjugated secondary antibodies for 1 hr at RT, before final washes. Protein bands were detected using ECL Chemiluminescent Substrate Reagent Kit (Invitrogen) and visualized on a Chemidoc XRS System and Analysis Software (Bio-Rad Laboratories).

### Primary cardiomyocytes

Neonatal ventricular cardiomyocytes were isolated from mixed sex, two-day-old C57BL6J mice^79^. Cells were seeded into Seahorse V7 assay plates at 60,000 cells/well. Prior to assays, cells were exposed to Aβ_42_ at 0, 200 and 300pM and co-incubated with ScrAβ_42_ at 300, 100 and 0pM, respectively, such that all cells were exposed to 300pM of total peptide for 48 hr, with media replenished every 24 hr. These supraphysiological Aβ_42_ concentrations were used as we measured Aβ_42_ levels in FBS containing media at ~20pM. Cells were washed and incubated in 600 µL assay running media (unbuffered DMEM, Invitrogen; supplemented with 25 mM glucose, 1 mM pyruvate, pH 7.4) in a non-CO_2_ incubator at 37 °C for 1 h before commencing the assay. Mitochondrial function was analysed by performing three baseline oxygen consumption rate (OCR) measurements, before subsequent measurements following injections of oligomycin (1 mM final concentration), carbonyl cyanide-p-trifluoromethoxyphenylhydrazone (FCCP; 1 μM final concentration), rotenone (1 μM final concentration) and Antimycin A (1 μM final concentration). Each measurement cycle consisted of the following: 3 min mix, 3 min wait, 3 min measure. Basal OCR was calculated by subtracting the mean value of the OCR measurements following Antimycin A injection from the mean of the baseline measurements. Maximal OCR was calculated by subtracting the mean value of the OCR measurements following Antimycin A injection from the mean value of the OCR measurements following FCCP injection^80^. Complex I-mediated respiration was designated as the difference between basal and rotenone (1μM)-suppressed respiration, which was also normalised by the maximal respiratory rate under FCCP stimulated conditions to determine flux as a percentage of maximal capacity. Cell viability was assessed by measuring extracellular and intracellular lactate dehydrogenase (LDH) following exposure of cardiomyocytes to 300pM of ScrAβ_42_ or Aβ_42_ for 48 hrs. LDH was measured using the CytoTox 96® Non-Radioactive Cytotoxicity Assay kit (Promega) as per manufacturer’s instructions. Viability was expressed as LDH in the media normalised to total LDH from the media and cell lysate^76^.

### Statistical analyses

All data are presented as mean ± SEM. Individual data points identified as greater than two standard deviations away from the mean were designated as outliers and removed. Data were assessed for normality using a Shapiro-Wilks test. Normally distributed data were analysed by independent samples t-test, one-way ANOVA, two-way ANOVA, two-way repeated measures ANOVA or mixed effects model, as described for statistically significant data in relevant figure legends. Data not normally distributed were analysed by Mann-Whitney test or Kruskal-Wallis test, as described for statistically significant data in relevant figure legends. All statistical analyses were performed using GraphPad Prism 9, with p values < 0.05 considered significant.

### Reporting summary

Further information on research design is available in the Nature Portfolio Reporting Summary linked to this article.

### Supplementary information

Supplementary information

Peer Review File

Reporting Summary

### Source data

Source data

## Data Availability

RNA-seq data generated in this study can be found at Gene Expression Omnibus, submission GSE213708. All other data generated during this study are provided in the Source Data file. No previously published datasets were used in this work. Source data are provided with this paper.

## Code availability

Scripts used for differential gene expression, enrichment analysis and mitch, as well as specific instructions for how to reproduce our data, are provided at the following link (https://github.com/markziemann/2dpw4liam). Code is also accessible and citable at 10.5281/zenodo.10203716.
