# Supplementary material for: Amyloid beta 42 alters cardiac metabolism and impairs cardiac function in male mice with obesity
Source: Nat Commun. 2024 Jan 15;15:258. doi: 10.1038/s41467-023-44520-4 (PMC10789867; doi:10.1038/s41467-023-44520-4)
Supplement: Supplementary file 1 — Supplementary information [file 41467_2023_44520_MOESM1_ESM.pdf]

Supplementary Information for

Amyloid beta 42 alters cardiac metabolism and impairs cardiac function in obese male mice

Liam G Hall[1,2,10], Juliane K. Czeczor[1,3,10], Timothy Connor[1], Javier Botella[1], Kirstie A. De Jong[1,4], Mark C. Renton[5], Amanda J. Genders[1,6], Kylie Venardos[1], Sheree D. Martin[1], Simon T. Bond[1,7], Kathryn Aston-Mourney[1], Kirsten F. Howlett[5], James A Campbell[8], Greg R. Collier[8], Ken R. Walder[1], Matthew McKenzie[9], Mark Ziemann[9], Sean L. McGee[1,8]

[1]Institute for Mental and Physical Health and Clinical Translation, Metabolic Research Unit, School of Medicine, Deakin University, Geelong, Australia.
[2]Department of Cellular and Physiological Sciences, Faculty of Medicine, The University of British Columbia, Vancouver, Canada.
[3]Becton Dickinson GmbH, Medical Affairs, 69126 Heidelberg, Germany.
[4]Institute of Experimental Cardiovascular Research, University Medical Centre Hamburg-Eppendorf, Hamburg, Germany.
[5]Institute for Physical Activity and Nutrition, School of Exercise and Nutrition Sciences, Deakin University, Geelong, Australia.
[6]Department of Nutrition, Dietetics and Food, School of Clinical Sciences and Victorian Heart Institute, Monash University, Melbourne, Australia
[7]Baker Heart and Diabetes Institute, Melbourne, Australia.
[8]Ambetex Pty Ltd, Geelong, Australia.
[9]School of Life and Environmental Science, Deakin University, Geelong, Australia.
[10]These authors contributed equally.

This document contains 5 data tables and 7 data figures.

Corresponding author:

Professor Sean L. McGee
Metabolic Remodelling Laboratory
Metabolic Research Unit
School of Medicine
Deakin University, Waurn Ponds, Australia, 3216
Ph: +61 3 5227 2519
Fax: +61 3 5227 2945
Email: sean.mcgee@deakin.edu.au

Supplementary Table 1

**Parameter**	**ScrAβ_{42}**	**Aβ_{42}**	***P* value**
Peak aortic flow (cm/sec)	61.9 ± 3.8	58.7 ± 1.1	0.481
Ejection time (msec)	52.5 ± 1.6	50.3 ± 2.0	0.411
Heart rate (bpm)	386 ± 24	397 ± 18	0.736
Peak E wave (cm/sec)	58.9 ± 4.9	60.6 ± 2.7	0.770
Peak A wave (cm/sec)	27.4 ± 2.4	32.0 ± 0.6	0.123
IVSd (cm)	0.126 ± 0.007	0.121 ± 0.006	0.604
LVIDd (cm)	0.346 ± 0.006	0.346 ± 0.016	0.974
LVPWd (cm)	0.108 ± 0.005	0.116 ± 0.004	0.155
IVSs (cm)	0.148 ± 0.007	0.149 ± 0.006	0.949
LVIDs (cm)	0.230 ± 0.003	0.251 ± 0.013	0.151
LVPWs (cm)	0.132 ± 0.006	0.148 ± 0.007	0.100
Estimated LV mass (mg)	163 ± 13	168 ± 14	0.822
Heart weight/tibia length (mg/mm)	7.11 ± 0.09	7.19 ± 0.17	0.751

Supplementary Table 1: Cardiac function and morphology in mice administered ScrAβ_{42} or Aβ_{42}. IVSd, intraventricular septum thickness at diastole; LVIDd, left ventricular internal diameter at diastole; LVPWd, left ventricular posterior wall thickness at diastole; IVSs, intraventricular septum thickness at systole; LVIDs, left ventricular internal diameter at systole; LVPWs, left ventricular posterior wall thickness at systole. Data are mean ± SEM, n =10 mice/group. Groups compared by unpaired t-test, two-tailed. Source data are provided in the Source Data file.

Supplementary Table 2

Parameter	ScrAβ_{40}	Aβ_{40}	*P* value
Peak aortic flow (cm/sec)	60.4 ± 4.2	66.2 ± 5.1	0.391
Ejection time (msec)	59.3 ± 1.4	61.5 ± 1.2	0.233
Heart rate (bpm)	416 ± 12	384 ± 10	0.060
Peak E wave (cm/sec)	57.1 ± 3.6	58.1 ± 4.4	0.862
Peak A wave (cm/sec)	35.9 ± 1.8	32.4 ± 1.8	0.193
E:A ratio	1.54 ± 0.08	1.73 ± 0.10	0.153
Deceleration time (msec)	0.032 ± 0.001	0.032 ± 0.001	0.939
IVSd (cm)	0.125 ± 0.005	0.120 ± 0.006	0.498
LVIDd (cm)	0.340 ± 0.010	0.350 ± 0.012	0.528
LVPWd (cm)	0.125 ± 0.004	0.128 ± 0.003	0.533
IVSs (cm)	0.178 ± 0.007	0.174 ± 0.007	0.697
LVIDs (cm)	0.225 ± 0.012	0.226 ± 0.013	0.981
LVPWs (cm)	0.141 ± 0.004	0.152 ± 0.005	0.094
Ejection fraction (%)	71.5 ± 2.6	73.2 ± 2.1	0.602
Fractional shortening (%)	35.9 ± 2.2	36.2 ± 1.7	0.905
Estimated LV mass (mg)	217 ± 15	204 ± 11	0.502
Heart weight/tibia length (mg/mm)	9.60 ± 0.42	8.78 ± 0.24	0.112

Supplementary Table 2: Cardiac function and morphology in mice administered ScrAβ_{40} or Aβ_{40}. IVSd, intraventricular septum thickness at diastole; LVIDd, left ventricular internal diameter at diastole; LVPWd, left ventricular posterior wall thickness at diastole; IVSs, intraventricular septum thickness at systole; LVIDs, left ventricular internal diameter at systole; LVPWs, left ventricular posterior wall thickness at systole. Data are mean ± SEM, n =12 mice/group. Groups compared by unpaired t-test, two-tailed. Source data are provided in the Source Data file.

Supplementary Table 3

	Control		3D6		2-way RM ANOVA *P* value		
**Parameter**	**Pre**	**Post**	**Pre**	**Post**	**Tx**	**Time**	**Int.**
Peak aortic flow (cm/sec)	72.2 ± 6.9	67.9 ± 4.7	67.0 ± 6.0	65.3 ± 4.7	0.5022	0.5975	0.8217
Ejection time (msec)	48.5 ± 2.3	54.0 ± 2.2	49.4 ± 3.2	52.5 ± 2.4	0.9121	0.0969	0.6365
Heart rate (bpm)	455 ± 22	453 ± 19	437 ± 17	475 ± 22	0.9328	0.3786	0.3177
Peak E wave (cm/sec)	48.3 ± 2.7	51.0 ± 3.8	51.1 ± 4.6	49.4 ± 3.3	0.8070	0.7936	0.3434
Peak A wave (cm/sec)	28.7 ± 1.6	37.4 ± 3.5	32.7 ± 3.1	35.0 ± 2.8	0.9631	0.1365	0.1724
E:A ratio	1.69 ± 0.08	1.41 ± 0.04	1.60 ± 0.09	1.32 ± 0.04	0.1654	0.0056	0.8433
IVSd (cm)	0.116 ± 0.003	0.128 ± 0.006	0.130 ± 0.006	0.129 ± 0.006	0.2207	0.1432	0.0829
LVIDd (cm)	0.272 ± 0.012	0.307 ± 0.009	0.272 ± 0.010	0.293 ± 0.013	0.3505	0.0072	0.7772
LVPWd (cm)	0.120 ± 0.006	0.135 ± 0.008	0.130 ± 0.007	0.131 ± 0.005	0.9798	0.0013	0.2343
IVSs (cm)	0.165 ± 0.002	0.174 ± 0.006	0.168 ± 0.007	0.166 ± 0.006	0.6910	0.5835	0.3740
LVIDs (cm)	0.175 ± 0.012	0.189 ± 0.010	0.177 ± 0.011	0.195 ± 0.012	0.7513	0.1800	0.8458
LVPWs (cm)	0.137 ± 0.006	0.158 ± 0.009	0.146 ± 0.005	0.148 ± 0.010	0.9417	0.1486	0.2658
Ejection fraction (%)	75.3 ± 1.5	75.0 ± 1.8	76.1 ± 1.8	69.5 ± 2.8	0.1797	0.3923	0.5001
Fractional shortening (%)	37.7 ± 1.3	37.6 ± 1.6	37.3 ± 1.9	34.6 ± 2.4	0.7223	0.1921	0.1953

Supplementary Table 3: Cardiac function and morphology in mice fed a high fat diet and administered control or 3D6 antibodies. IVSd, intraventricular septum thickness at diastole; LVIDd, left ventricular internal diameter at diastole; LVPWd, left ventricular posterior wall thickness at diastole; IVSs, intraventricular septum thickness at systole; LVIDs, left ventricular internal diameter at systole; LVPWs, left ventricular posterior wall thickness at systole. Data are mean ± SEM, n =12 mice/group. Groups compared by two-way repeated measures ANOVA. Tx, treatment; Int, interaction. Source data are provided in the Source Data file.

Supplementary Table 4

	Chow control			HFD control			HFD 3D6			Mixed effects model *P* value		
	Base-line	Pre-treat	Post-treat	Base-line	Pre-treat	Post-treat	Base-line	Pre-treat	Post-treat	Tx	Time	Int.
PAF (cm/sec)	57.8 ± 2.7	59.1 ± 4.2	61.3 ± 2.6	60.1 ± 6.3	59.3 ± 3.2	67.7 ± 5.1	58.7 ± 4.0	64.1 ± 3.0	71.8 ± 2.5	0.0955	0.0067	0.5443
ET (msec)	42.3 ± 1.9	42.3 ± 2.2	46.8 ± 2.0	41.2 ± 1.2	49.2 ± 2.4	43.8 ± 3.2	44.5 ± 1.5	46.2 ± 1.5	45.3 ± 2.6	0.6138	0.0318	0.5453
HR (bpm)	468 ± 18	491 ± 17	510 ± 23	441 ± 4	501 ± 9	556 ± 23	462 ± 10	503 ± 12	572 ± 12	0.3321	< 0.0001	0.3321
Peak E (cm/sec)	42.3 ± 3.5	51.1 ± 3.8	50.0 ± 4.1	45.2 ± 4.2	58.3 ± 4.1	53.0 ± 2.6	41.6 ± 1.4	52.3 ± 2.8	63.8 ± 3.5	0.2964	0.0003	0.2945
Peak A (cm/sec)	27.3 ± 2.2	33.5 ± 2.7	35.0 ± 1.9	24.4 ± 1.2	34.4 ± 2.4	36.2 ± 2.8	28.7 ± 1.7	32.3 ± 2.2	46.7 ± 3.8	0.0852	< 0.0001	0.0592
E:A ratio	1.62 ± 0.08	1.59 ± 0.03	1.57 ± 0.03	1.65 ± 0.05	1.67 ± 0.06	1.45 ± 0.09	1.52 ± 0.05	1.56 ± 0.07	1.41 ± 0.06	0.2417	0.1167	0.8675
IVSd (cm)	0.147 ± 0.002	0.109 ± 0.005	0.118 ± 0.004	0.135 ± 0.006	0.114 ± 0.003	0.129 ± 0.005	0.137 ± 0.008	0.125 ± 0.005	0.122 ± 0.003	0.7234	< 0.0001	0.0335
LVIDd (cm)	0.248 ± 0.011	0.314 ± 0.014	0.293 ± 0.014	0.268 ± 0.005	0.311 ± 0.015	0.308 ± 0.015	0.263 ± 0.014	0.303 ± 0.014	0.281 ± 0.009	0.4968	0.0001	0.6252
LVPWd (cm)	0.156 ± 0.008	0.136 ± 0.008	0.125 ± 0.008	0.142 ± 0.005	0.152 ± 0.007	0.137 ± 0.009	0.134 ± 0.007	0.147 ± 0.008	0.144 ± 0.007	0.6857	0.0577	0.0241
IVSs (cm)	0.189 ± 0.008	0.158 ± 0.006	0.162 ± 0.006	0.175 ± 0.008	0.169 ± 0.005	0.178 ± 0.006	0.167 ± 0.009	0.169 ± 0.003	0.173 ± 0.004	0.9053	0.0876	0.0313
LVIDs (cm)	0.160 ± 0.007	0.193 ± 0.013	0.185 ± 0.015	0.192 ± 0.010	0.192 ± 0.016	0.185 ± 0.015	0.186 ± 0.012	0.188 ± 0.014	0.172 ± 0.010	0.4420	0.6350	0.6135
LVPWs (cm)	0.171 ± 0.005	0.156 ± 0.007	0.141 ± 0.008	0.160 ± 0.006	0.174 ± 0.010	0.166 ± 0.011	0.149 ± 0.009	0.167 ± 0.008	0.157 ± 0.008	0.2567	0.2109	0.0370
EF (%)	71.0 ± 1.9	76.1 ± 2.1	74.1 ± 2.9	67.3 ± 2.6	74.9 ± 3.0	78.2 ± 2.2	66.2 ± 1.6	74.3 ± 3.1	76.7 ± 2.2	0.8585	0.004	0.5063
FS (%)	34.0 ± 1.4	39.0 ± 2.1	37.5 ± 2.6	31.8 ± 1.8	39.5 ± 3.0	40.9 ± 2.3	30.0 ± 0.9	38.3 ± 2.9	39.4 ± 2.0	0.9689	0.0005	0.6390
LV mass (mg)	152 ± 7	153 ± 9	136 ± 3	161 ± 10	164 ± 5	161 ± 8	145 ± 14	166 ± 6	153 ± 5	0.0925	0.1080	0.1357

Supplementary Table 4: Cardiac function and morphology in mice fed chow or high fat diet and administered control or 3D6 antibodies. PAF, peak aortic flow; ET, ejection time; HR, heart rate; IVSd, intraventricular septum thickness at diastole; LVIDd, left ventricular internal diameter at diastole; LVPWd, left ventricular posterior wall thickness at diastole; IVSs, intraventricular septum thickness at systole; LVIDs, left ventricular internal diameter at systole; LVPWs, left ventricular posterior wall thickness at systole. EF, ejection fraction; FS, fractional shortening. Data are mean ± SEM, n =12 mice/group. Groups compared by mixed effects model. Tx, treatment; Int, interaction. Source data are provided in the Source Data file.

Supplementary Table 5

Pathway	setSize	p MANOVA	p.adjust MANOVA	s.dist	s.Aβ$_{42}$	s.3D6	p.Aβ$_{42}$	p.3D6
TCA cycle	22	<0.0001	0.0004	0.608	-0.350	0.498	0.0045	<0.0001
Pyruvate metabolism and TCA cycle	50	<0.0001	<0.0001	0.567	-0.305	0.478	0.0002	<0.0001
Pyruvate metabolism	26	0.0002	0.0030	0.484	-0.246	0.417	0.0301	0.0002
Mitochondrial biogenesis	71	0.0009	0.0014	0.268	-0.184	0.196	0.0076	0.0044
Protein localisation	141	0.0001	0.0004	0.215	-0.146	0.158	0.0028	0.0012
Neddylation	201	<0.0001	<0.0001	0.199	-0.109	0.166	0.0079	<0.0001
Antigen processing	262	<0.0001	<0.0001	0.197	-0.075	0.182	0.0380	<0.0001
Autophagy	117	0.0037	0.0050	0.188	-0.127	0.139	0.0178	0.0097
Chromatin modifying enzymes	188	0.0006	0.0011	0.171	-0.111	0.130	0.0002	0.0022
Chromatin organisation	188	0.0006	0.0011	0.171	-0.111	0.130	0.0002	0.0022
Hemostasis	373	<0.0001	<0.0001	0.184	0.094	-0.157	0.0019	<0.0001
Platelet activation	187	<0.0001	<0.0001	0.214	0.098	-0.190	0.0208	<0.0001
Elevated platelet cytosolic-Ca2+	97	0.0002	0.0005	0.252	0.148	-0.203	0.0117	0.0005
Platelet degranulation	93	0.0001	0.0003	0.269	0.158	-0.218	0.0084	0.0003
Chondroitin sulfate metabolism	40	0.0040	0.0050	0.319	0.205	-0.244	0.0076	0.0044
Kainate receptor activation	20	0.0052	0.0060	0.439	0.339	-0.278	0.0086	0.0314
Basigin interactions	18	0.0042	0.0291	0.473	0.343	-0.326	0.0118	0.0168
Thrombin signalling through PARs	21	0.0013	0.0116	0.480	0.336	-0.343	0.0076	0.0065
Gβχ signalling through BTK	11	0.0029	0.0021	0.508	0.371	-0.346	0.00331	0.0468

Supplementary Table 5: Reactome pathways reciprocally regulated in mice administered Aβ$_{42}$ or 3D6 relative to their respective control groups, as determined by mitch. MANOVA p-values (both unadjusted and adjusted for multiple comparison testing) are for pathways in two dimensions. s.dist is the enrichment score across two dimensions. s.Aβ$_{42}$ and s.3D6 are the enrichment scores for the Aβ$_{42}$ and 3D6 datasets respectively. p.Aβ$_{42}$ and p.3D6 indicate the significance of the pathway in the Aβ$_{42}$ and 3D6 datasets respectively and have been adjusted for multiple comparisons using the FDR method. Source data are provided in the Source Data file.

Supplementary Figure 1

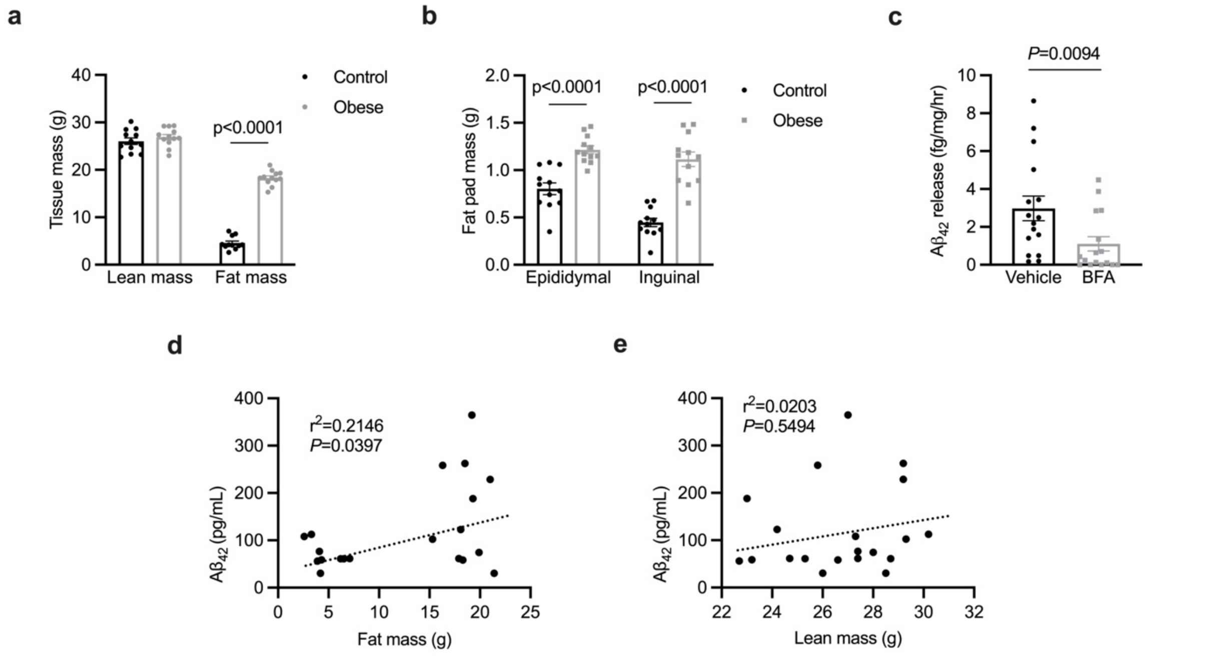

Supplementary Figure 1: Aβ_{42} release by adipose tissue and body composition correlations with plasma Aβ_{42}. a, lean and fat mass in control and obese mice (n=12/group). **b,** fat pad mass in control and obese mice (n=12/group). **c,** relative release of Aβ_{42} from adipose tissue exposed to vehicle or Brefeldin A (BFA; Mann-Whitney test, U=73; n=16/group). **d**, correlation between fat mass and plasma Aβ_{42} (Pearsons correlation coefficient test; n= 20 data points). **e**, Correlation between lean mass and plasma Aβ_{42} (Pearsons correlation coefficient test; n= 20 data points). All data are mean ± SEM. Statistical tests are two-tailed. Source data are provided in the Source Data file.

Supplementary Figure 2

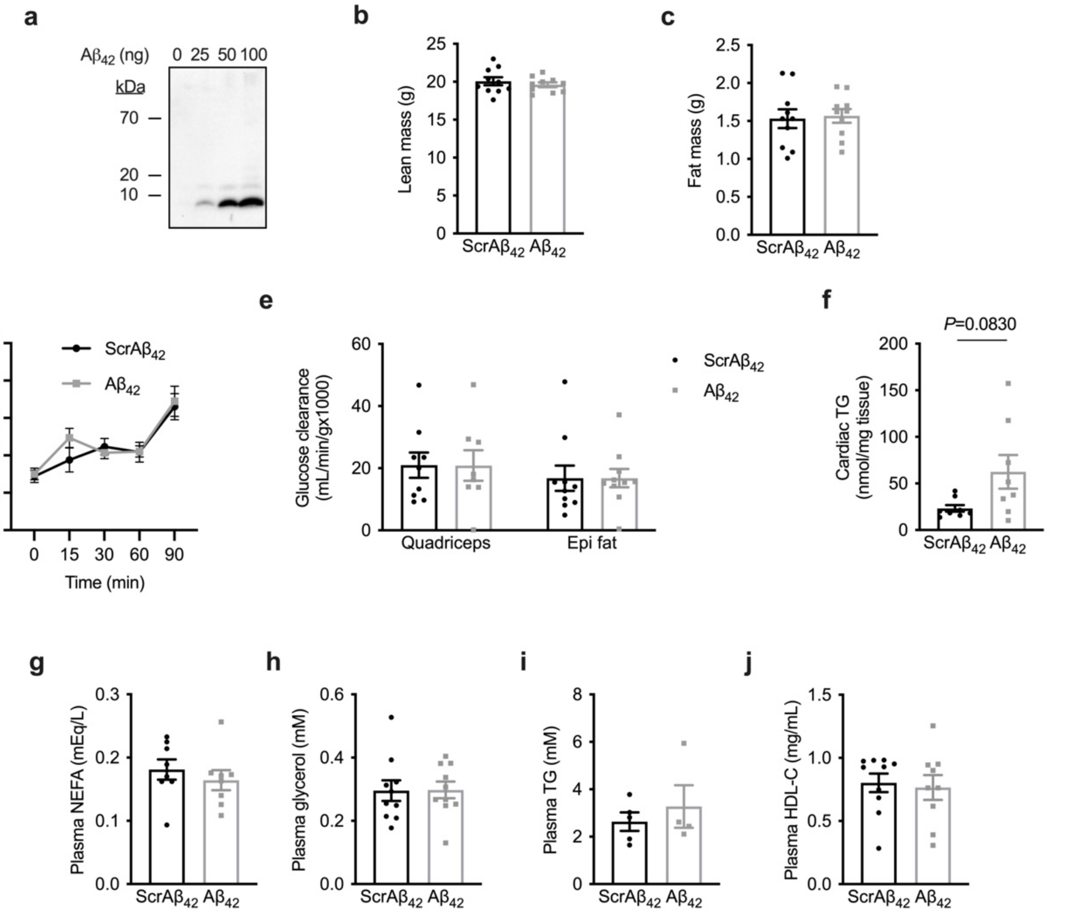

Supplementary Figure 2: Characterisation of mice administered ScrAβ_{42} or Aβ_{42}. a, immunoblotting of increasing amounts of recombinant Aβ_{42} preparations. **b,** lean mass (n=10/group); **c**, fat mass (n=10/group); **d**, plasma insulin during a glucose tolerance test (n=9 and 10/group respectively); **e** glucose clearance by the quadriceps skeletal muscle (n=9 and 8/group respectively) and epididymal fat pad (n=10/group); **f**, ^{14}C-glucose incorporation into cardiac lipids (n=8/group); **g**, plasma non-esterified fatty acids (NEFA; n=8/group); **h**, plasma glycerol (n=10/group); **i**, plasma triglycerides (TG; n=5 and 4/group respectively), and; **j**, plasma high-density lipoprotein cholesterol (HDL-C; n=10 and 9/group respectively) in mice administered ScrAβ_{42} or Aβ_{42}. All data are mean ± SEM. Statistical tests are two-tailed. Source data are provided in the Source Data file.

Supplementary Figure 3

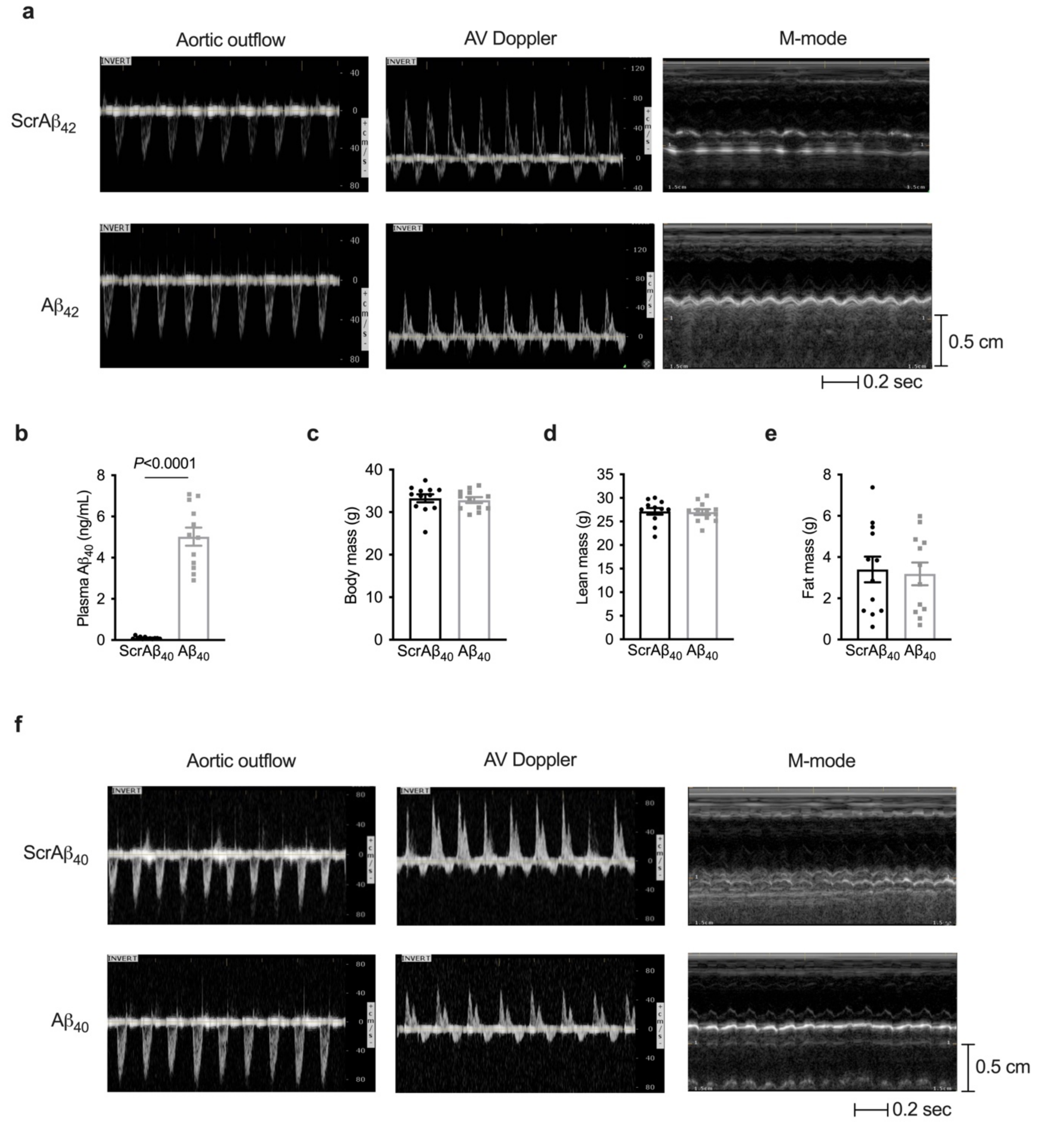

Supplementary Figure 3: Characterisation of mice administered ScrAβ_{42} or Aβ_{42} and ScrAβ_{40} or Aβ_{40}. a, representative echocardiography images of mice administered ScrAβ_{42} or Aβ_{42}. **b**, plasma Aβ_{40} 60 min after Aβ_{40} administration (n=12/group). **c**, body weight (n=12/group); **d**, lean mass (n=12/group); **e**, fat mass (n=12/group), and; **f**, representative echocardiography images in mice administered ScrAβ_{40} or Aβ_{40}. All data are mean ± SEM. Statistical tests are two-tailed. Source data are provided in the Source Data file.

Supplementary Figure 4

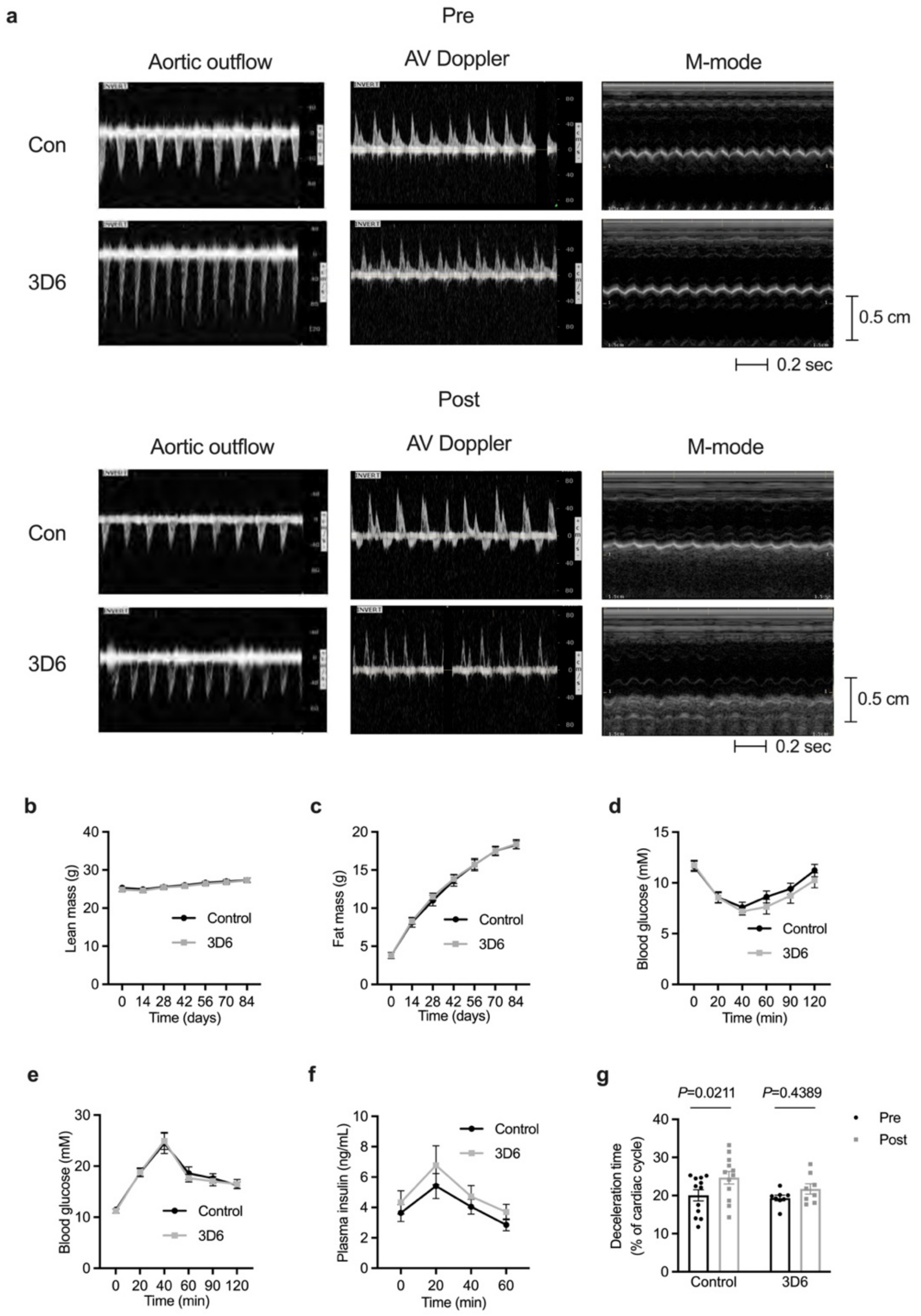

Supplementary Figure 4: Characterisation of mice fed a high fat diet and administered control or 3D6 antibodies. **a**, representative echocardiography images; **b**, lean mass (n=12/group); **c**, fat mass (n=12/group), **d**, blood glucose during the insulin tolerance test (n=12/group); **e**, blood glucose during the glucose tolerance test (n=11 and 12/group respectively); **f**, plasma insulin during the glucose tolerance test (n=9 and 10/group respectively), and; **g**, cardiac deceleration time expressed as a percentage of the cardiac cycle (mixed effects model (time $P = 0.0140$, $F(1,18) = 7.407$) with Sidak's repeated measures test *P*.adjusted; n=12 and 8/group respectively) pre- and post-treatment in mice fed a high fat diet and administered control and 3D6 antibodies. All data are mean ± SEM. Statistical tests are two-tailed. Source data are provided in the Source Data file.

Supplementary Figure 5

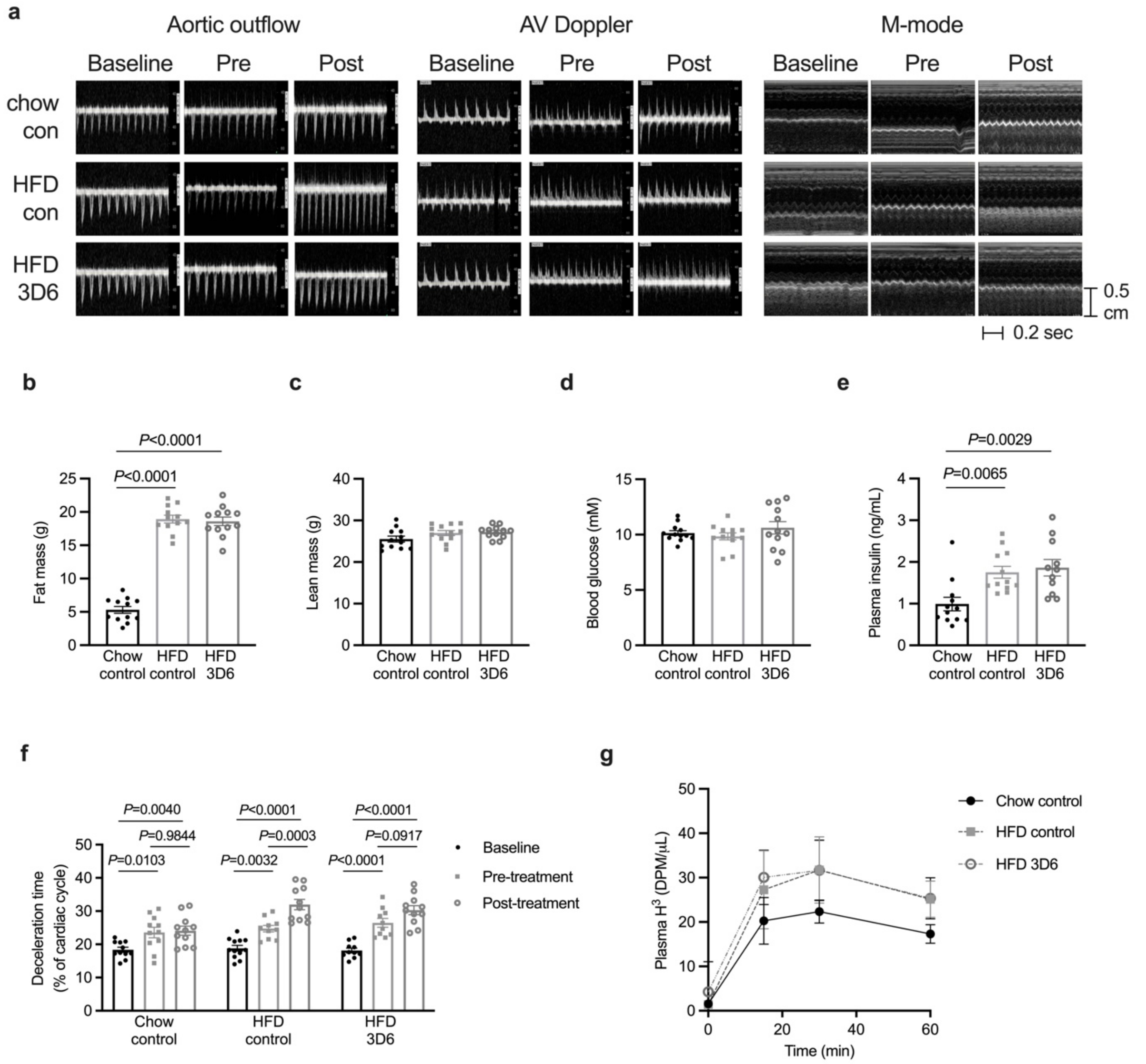

Supplementary Figure 5: Characterisation of mice fed a high fat diet and administered control or 3D6 antibodies. **a**, representative echocardiography images; **b**, fat mass (one-way ANOVA ($P < 0.0001$; F(2,33) = 177.6) with Sidak's repeated measures test *P*.adjusted; n=12/group); **c**, lean mass (n=12/group), **d**, fasting blood glucose (n=12/group); **e**, fasting plasma insulin (Kruskal-Wallis test ($P = 0.0011$; $X^2 = 13.66$) with Dunn's repeated measures test *P*.adjusted; n=12, 12 and 11/group respectively). **f**, cardiac deceleration time expressed as a percentage of the cardiac cycle (mixed effects model (time $P < 0.0001$, $F(2,56) = 56.37$; treatment $P = 0.0044$, $F(2,32) = 6.445$; interaction $P = 0.0145$, $F(4,56) = 3.410$) with Sidak's repeated measures test *P*.adjusted; n=12/group); **g,** plasma ^3H concentration in mice fed regular chow and administered control antibody, or mice fed a high fat diet (HFD) and administered control or 3D6 antibodies (n=7, 8 and 8/group respectively). All data are mean ± SEM. Statistical tests are two-tailed. Source data are provided in the Source Data file.

Supplementary Figure 6

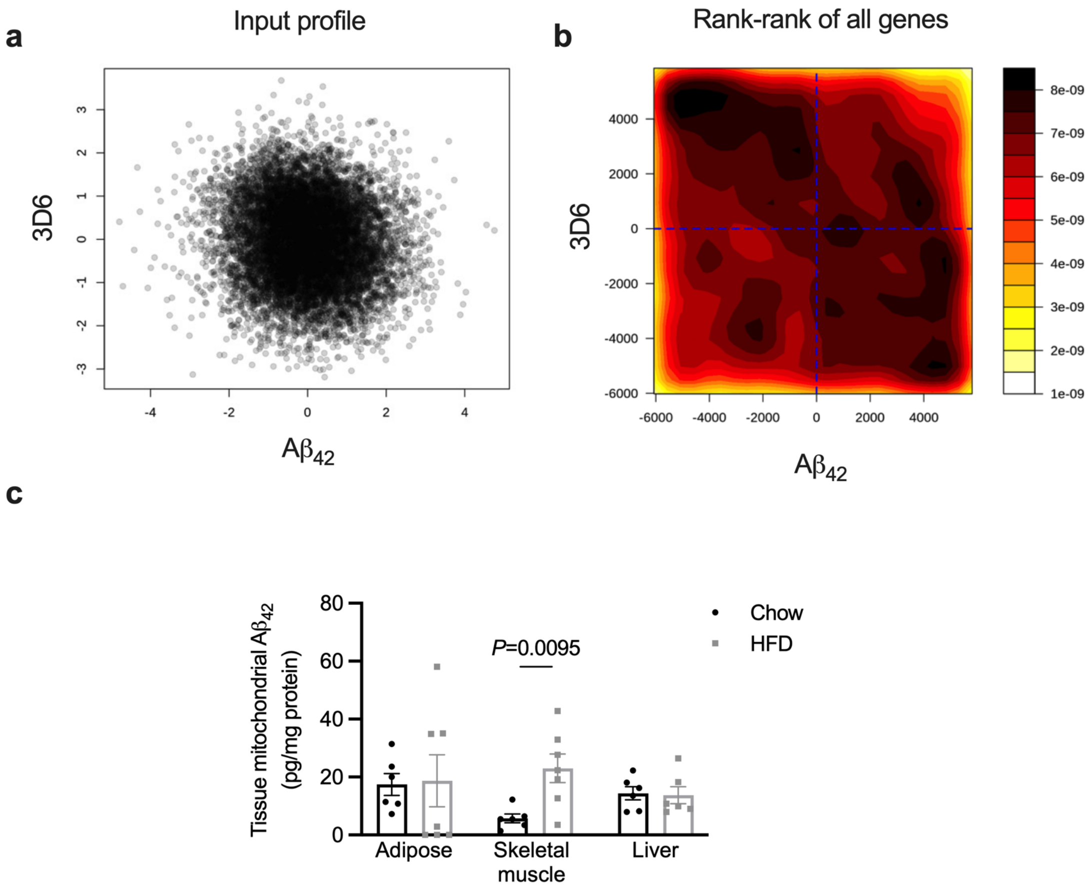

Supplementary Figure 6: Mechanisms by which Aβ_{42} has its effects on the heart. a, input profile defined as differential expression score defined as the sign of the fold change multiplied by the -log10(p-value); **b**, gene ranks from bulk RNA-seq analysis of gene expression in the hearts of mice administered Aβ_{42} compared with mice administered ScrAβ_{42} (x-axis) and hearts of mice administered 3D6 antibody compared with mice administered control antibody (y-axis). **c,** Aβ_{42} in mitochondrial fractions isolated from adipose tissue, skeletal muscle and liver of chow or HFD-fed mice (unpaired t-test; n=6 and 7/group respectively). All data are mean ± SEM. Statistical tests are two-tailed. Source data are provided in the Source Data file.

Supplementary Figure 7

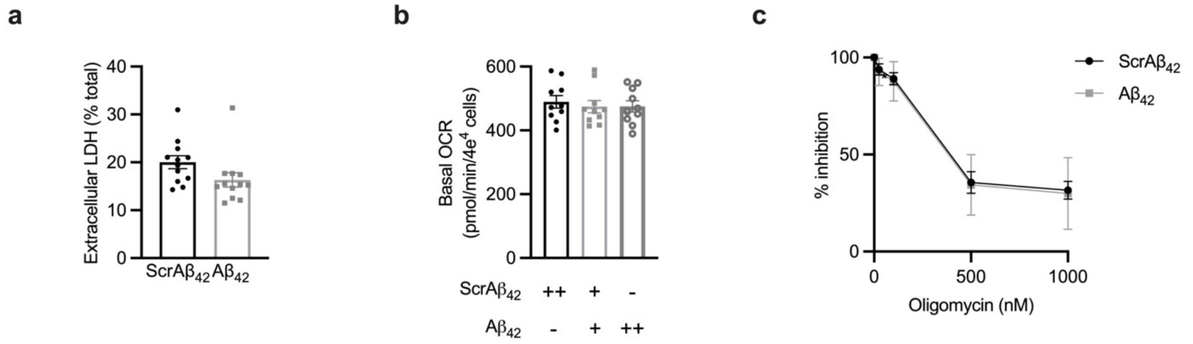

Supplementary Figure 7: Effect of Aβ42 on cardiomyocytes. a, extracellular lactate dehydrogenase (LDH) in primary neonatal ventricular cardiomyocytes (NVCM) exposed to ScrAb42 or Ab42 for 48 hr (n=12 biological replicates/group). **b,** basal oxygen consumption rate (OCR) in FAO hepatocytes exposed to ScrAβ42 or Aβ42 (ScrAβ42 at 300, 100 and 0pM and co-incubated with Aβ42 at 0, 200 and 300pM) for 48 hrs with glucose and pyruvate as exogenous substrates (n=10 biological replicates/group). **c,** inhibition of respiration in response to increasing concentrations of oligomycin in primary NVCM exposed to ScrAβ42 or Aβ42 for 48 hrs (n=5 biological replicates/group). All data are mean ± SEM. Source data are provided in the Source Data file.
