# Supplementary material for: Amyloid beta 42 alters cardiac metabolism and impairs cardiac function in male mice with obesity
Source: Nat Commun. 2024 Jan 15;15:258. doi: 10.1038/s41467-023-44520-4 (PMC10789867; doi:10.1038/s41467-023-44520-4)
Supplement: Supplementary file 3 — Reporting Summary [file 41467_2023_44520_MOESM3_ESM.pdf]

nature portfolio

Corresponding author(s): Sean McGee

Last updated by author(s): 15.12.2023

Reporting Summary

Nature Portfolio wishes to improve the reproducibility of the work that we publish. This form provides structure for consistency and transparency in reporting. For further information on Nature Portfolio policies, see our Editorial Policies and the Editorial Policy Checklist.

Statistics

For all statistical analyses, confirm that the following items are present in the figure legend, table legend, main text, or Methods section.

n/a	Confirmed	
☐	☒	The exact sample size (*n*) for each experimental group/condition, given as a discrete number and unit of measurement
☐	☒	A statement on whether measurements were taken from distinct samples or whether the same sample was measured repeatedly
☐	☒	The statistical test(s) used AND whether they are one- or two-sided<br>*Only common tests should be described solely by name; describe more complex techniques in the Methods section.*
☒	☐	A description of all covariates tested
☐	☒	A description of any assumptions or corrections, such as tests of normality and adjustment for multiple comparisons
☐	☒	A full description of the statistical parameters including central tendency (e.g. means) or other basic estimates (e.g. regression coefficient) AND variation (e.g. standard deviation) or associated estimates of uncertainty (e.g. confidence intervals)
☐	☒	For null hypothesis testing, the test statistic (e.g. *F*, *t*, *r*) with confidence intervals, effect sizes, degrees of freedom and *P* value noted<br>*Give P values as exact values whenever suitable.*
☒	☐	For Bayesian analysis, information on the choice of priors and Markov chain Monte Carlo settings
☒	☐	For hierarchical and complex designs, identification of the appropriate level for tests and full reporting of outcomes
☒	☐	Estimates of effect sizes (e.g. Cohen's *d*, Pearson's *r*), indicating how they were calculated

Our web collection on statistics for biologists contains articles on many of the points above.

Software and code

Policy information about availability of computer code

Data collection	Echocardiography images were capture and quantified using the Phillips HD15 ultrasound system and software (revision 3.0). Body composition was measured using EchoMRI 500 and software (EchoMRI). PCR reactions were quantified using QuantStudio3 software (Applied Biosystems). Assays requiring colorimetric or fluorescence quantification used the Victor Nivo multi-modal plate reader and Victor Nivo software (version 4.5; Perkin Elmer). Western blots were scanned using the ChemiDoc MP Imaging System and Image Lab software (BioRad). Radioisotopes were quantified using a TriCarb 2800TR Liquid Scintillation Analyzer and QuantaSmart software (Perkin Elmer). Cellular respiration was captured with the Seahorse XFe24 Analyzer and Wave software (version 2.6.1.56; Agilent Technologies). RNA-seq was performed using the NovaSeq 6000 and control software (version 1.7; Illumina).
Data analysis	RNA-seq counts were read into R (version 4.0.2). Differential gene expression analysis was performed with DESeq2 (versionl.28.1). Multi-contrast enrichment analysis was performed with mitch (version 1.0.6). Western blots were quantified with Image Lab software (BioRad) GraphPad Prism 9 was used for statistical analyses. Scripts used for differential gene expression, enrichment analysis and mitch, as well as specific instructions for how to reproduce our data, are provided at the following link (https://github.com/markziemann/2dpw4liam). Code is also accessible and citable at DOI: 10.5281/zenodo.10203716.

For manuscripts utilizing custom algorithms or software that are central to the research but not yet described in published literature, software must be made available to editors and reviewers. We strongly encourage code deposition in a community repository (e.g. GitHub). See the Nature Portfolio guidelines for submitting code & software for further information.

Data

Policy information about availability of data

All manuscripts must include a data availability statement. This statement should provide the following information, where applicable:

- Accession codes, unique identifiers, or web links for publicly available datasets
- A description of any restrictions on data availability
- For clinical datasets or third party data, please ensure that the statement adheres to our policy

RNA-seq data generated in this study can be found at Gene Expression Omnibus, submission GSE213708. All other data generated during this study are provided in the Source Data file.

Research involving human participants, their data, or biological material

Policy information about studies with human participants or human data. See also policy information about sex, gender (identity/presentation), and sexual orientation and race, ethnicity and racism.

Reporting on sex and gender	N/A
Reporting on race, ethnicity, or other socially relevant groupings	N/A
Population characteristics	N/A
Recruitment	N/A
Ethics oversight	N/A

Note that full information on the approval of the study protocol must also be provided in the manuscript.

Field-specific reporting

Please select the one below that is the best fit for your research. If you are not sure, read the appropriate sections before making your selection.

☒ Life sciences ☐ Behavioural & social sciences ☐ Ecological, evolutionary & environmental sciences

For a reference copy of the document with all sections, see nature.com/documents/nr-reporting-summary-flat.pdf

Life sciences study design

All studies must disclose on these points even when the disclosure is negative.

Sample size	No statistical method was used to determine sample size. Sample sizes were chosen based on studies with similar experimental design, the outcome measure and the known variability of the outcome measure (Gaur et al. Diabetes, Obesity and Metabolism, 2016). For example, some measures of cardiac function can be difficult to obtain in obese mice and therefore groups of up to 16 mice were analysed.
Data exclusions	Echocardiography images that could not be properly interpreted were excluded from analyses. For real time PCR analyses, samples that had no amplification the gene of interest were excluded from analysis. For ELISA analyses, samples with values that were below background were excluded from analysis. Individual data points identified as greater than two standard deviations away from the mean were designated as outliers and removed.
Replication	All experiments were performed between 2 and 4 times and all data generated were included in the presented data. Replicates represent biological replicates. Mice used for experiments were obtained from multiple different litters.
Randomization	Groups were randomly assigned except for experiments with repeat echocardiography measures. In these experiments, mice were assigned to groups so that the primary outcome measures (deceleration time) was matched between groups prior to treatment interventions.
Blinding	Electrocardiography images were analyzed in a blinded manner. Investigators administering peptides and antibodies were aware of mouse groupings. Investigators handling mice and mouse samples aware of mouse code numbers but not code number group allocations.

Reporting for specific materials, systems and methods

We require information from authors about some types of materials, experimental systems and methods used in many studies. Here, indicate whether each material, system or method listed is relevant to your study. If you are not sure if a list item applies to your research, read the appropriate section before selecting a response.

Materials & experimental systems

n/a	Involved in the study
☐	☒ Antibodies
☒	☐ Eukaryotic cell lines
☒	☐ Palaeontology and archaeology
☐	☒ Animals and other organisms
☒	☐ Clinical data
☒	☐ Dual use research of concern
☒	☐ Plants

Methods

n/a	Involved in the study
☒	☐ ChIP-seq
☒	☐ Flow cytometry
☒	☐ MRI-based neuroimaging

Antibodies

Antibodies used

Anti-CoxIV, Cell Signalling, #4844
Anti-H4, Cell Signalling, #2592
Anti-p38 MAPK, Cell Signalling, #9212
Anti-PDH E1a, Cell Signalling, #2784
Anti-SDHa, Cell Signalling, #11998
Anti-a-tubulin, Sigma Aldrich, #T6074
Anti-rabbit IgG, HRP linked, Cell Signalling #7074
Anti-mouse IgG, HRP-linked, Cell Signalling #7076

Validation

All the antibodies were validated by the manufacturer:

Anti-CoxIV, Cell Signalling, #4844
Rabbit polyclonal antibody detects endogenous levels of total COX IV protein with an epitope corresponding to sequences surrounding Lys29 of human COX IV.
Suitable for western blotting, immunoprecipitation and immunohistochemistry.
Reacts with human, mouse, rat, monkey and bovine.

Anti-H4, Cell Signalling, #2592
Rabbit polyclonal antibody detects endogenous levels of total histone H4 protein with an epitope corresponding to the amino-terminal sequence of human histone H4. The antibody does not cross-react with other histones.
Suitable for western blotting.
Reacts with human, mouse, rat, monkey, D. melanogaster, zebrafish and S. cerevisiae.

Anti-p38 MAPK, Cell Signalling, #9212
Rabbit polyclonal antibody detects endogenous levels of total p38α, -β or -γ MAPK protein with an epitope corresponding to the sequence of human p38 MAPK. This antibody does not recognize p38δ, JNK/SAPK or p44/42 MAPK.
Suitable for western blotting.
Reacts with human, mouse, rat, monkey and guinea pig.

Anti-PDH E1a, Cell Signalling, #2784
Rabbit polyclonal antibody detects endogenous levels of total α1 and α2 subunits of pyruvate dehydrogenase protein with an epitope corresponding to sequences of both α1 and α2 subunits of human pyruvate dehydrogenase.
Suitable for western blotting.
Reacts with human, mouse, rat and monkey.

Anti-SDHa, Cell Signalling, #11998
Rabbit monoclonal antibody detects endogenous levels of total SDHA protein with an epitope corresponding to sequences surrounding Gly166 of human SDHA protein.
Suitable for western blotting, immunoprecipitation, immunohistochemistry and immunofluorescence.
Reacts with human, mouse, rat, monkey and hamster.

Anti-Troponin, Cell Signalling, #4002
Rabbit polyclonal antibody detects endogenous levels of total troponin I, both skeletal muscle and cardiac isoforms, with an epitope corresponding to the sequence of human human troponin I.
Suitable for western blotting, immunohistochemistry, immunofluorescence and flow cytometry.
Reacts with human, mouse and rat.

Anti-a-tubulin, Sigma Aldrich, #T6074
Monoclonal Anti-α-Tubulin (mouse IgG1 isotype) is derived from the hybridoma B-5-1-2 produced by the fusion of mouse myeloma cells and splenocytes from mice immunized with Sarkosyl-resistant filaments from Strongylocentrotus purpuratus (sea urchin).
Recognizes an epitope located at the C-terminal end of the α-tubulin isoform in a variety of organisms.
Suitable for western blotting.
Reacts with mouse, chicken, chlamydomonas, african green monkey, human, rat, bovine, sea urchin and kangaroo rat.

Animals and other research organisms

Policy information about studies involving animals; ARRIVE guidelines recommended for reporting animal research, and Sex and Gender in Research

Laboratory animals	For animal experiments, male C57BL6 mice were used and were obtained from the Australian Resource Centre. Mice were group housed with four to five mice per cage with a 12 hr light/dark cycle at 22oC with humidity 20-60%. Experiments commenced when mice were 10-12 weeks of age.
Wild animals	No wild animals were used.
Reporting on sex	Only male mice were used, as the cardiac response to high fat feeding has best been characterised in male mice.
Field-collected samples	No field-collected samples were used.
Ethics oversight	All procedures involving animals were approved by The Deakin University Animal Welfare Committee (approval numbers A58-2010, G07-2013, G15-2017 and G08-2020), which is subject to the Australian Code for the Responsible Conduct of Research.

Note that full information on the approval of the study protocol must also be provided in the manuscript.

Plants

Seed stocks	N/A
Novel plant genotypes	N/A
Authentication	N/A
